# Beta tACS of varying intensities differentially affect resting-state and movement-related sensorimotor power

**DOI:** 10.3389/fnins.2025.1524653

**Published:** 2025-06-04

**Authors:** Kym Wansbrough, Welber Marinovic, Hakuei Fujiyama, Ann-Maree Vallence

**Affiliations:** ^1^School of Psychology, College of Health and Education, Murdoch University, Perth, WA, Australia; ^2^Centre for Healthy Ageing, Health Futures Institute, Murdoch University, Perth, WA, Australia; ^3^Centre for Molecular Medicine and Innovative Therapeutics, Health Futures Institute, Murdoch University, Perth, WA, Australia; ^4^School of Population Health, Curtin University, Perth, WA, Australia

**Keywords:** transcranial alternating current stimulation, electroencephalography, neural oscillations, beta oscillations, motor cortex, motor control, power

## Abstract

Individuals who face difficulties with voluntary movement experience considerable challenges in performing everyday tasks, significantly compromising their sense of autonomy. Transcranial alternating current stimulation (tACS) holds promise in modulating sensorimotor beta oscillations, which underscore voluntary movement. However, the exact effect of beta tACS on oscillatory power is still largely elusive. This study aimed to examine the effect of different intensities of beta tACS (20 Hz) on both resting-state and event-related sensorimotor oscillations. Twenty-one healthy young adults (13 female; mean age 24.30 ± 4.84 years) received four separate 20 min sessions of tACS at different intensities (sham, 0.5 mA, 1.0 mA, or 1.5 mA, peak-to-peak), targeting the left primary motor cortex during rest. Electroencephalography (EEG) was recorded before and after stimulation, during both resting state and a self-paced right index finger button press task. Changes in sensorimotor beta power (13–30 Hz) were analyzed. For the resting-state, none of the real stimulation intensities induced significant changes in beta power relative to sham. For event-related activity, we observed intensity-dependent changes in bilateral broadband power (4–90 Hz): during movement preparation, 1.0 mA stimulation increased power; during movement termination, 0.5 mA stimulation decreased power while 1.0 mA and 1.5 mA stimulation induced comparable increases in power. While none of the stimulation intensities induced changes in broadband power during movement execution, 1.0 mA stimulation shifted participants’ peak beta frequency toward the tACS frequency. Interestingly, changes in power during movement preparation and execution following 1.0 mA stimulation were negatively associated with participants’ pre-tACS peak beta frequency. Together, these findings contribute to our understanding of the sensorimotor response to beta tACS, as well as the effect of stimulation intensity on tACS-induced neuromodulation, which has important implications for research and clinical settings.

## 1 Introduction

Voluntary movement is fundamental for carrying out daily activities and interacting with our environment. It is the very essence of our autonomy and expression as human beings. Unfortunately, many individuals struggle with movement-related issues, including older adults (Zapparoli et al., 2022), movement-disorder patients (Goetz et al., 2008), and patients recovering from stroke (Stinear et al., 2020). While existing treatment options offer some respite, it is imperative to develop novel approaches that not only yield additional advantages but also provide an alternative recourse when conventional methods prove ineffective.

The synchronous and rhythmic activity generated by neuronal populations, termed neural oscillations, have been suggested to play an important role in successful voluntary movement (Engel and Fries, 2010). Specifically, beta oscillations (13–30 Hz) have been implicated in voluntary movement, reflected in three robust movement-related changes over sensorimotor regions: (1) pre-movement beta event-related desynchronization (ERD), (2) movement beta ERD, and (3) post-movement beta event-related synchronization (ERS; for a review, see Kilavik et al., 2013). The beta ERD is thought to reflect the activation of motor areas for movement preparation and execution, and the movement ERS is thought to reflect motor inhibition (Kilavik et al., 2013). Further refining our understanding of these oscillatory patterns, researchers have identified functional distinctions between low beta (13–20 Hz) and high beta (20–30 Hz) sub-bands. Low beta is predominantly associated with movement preparation and post-movement inhibition, while high beta is more closely related to motor control, movement execution, and sensorimotor processing (Nougaret et al., 2024). In healthy adults, both resting-state and event-related sensorimotor beta oscillations have been associated with motor performance (Espenhahn et al., 2019). Further, in individuals with motor impairment (e.g., stroke patients), sensorimotor beta oscillations show abnormal activity (Rossiter et al., 2014), which has been used to predict improvements in motor performance (Espenhahn et al., 2020). Taken together, these studies suggest that sensorimotor beta oscillations play a key role in successful voluntary movement. Thus, there is much interest in developing techniques that can effectively and reliably modulate these oscillations to improve voluntary movement.

To modulate neural oscillations, there is growing interest in the use of transcranial alternating current stimulation (tACS)—a safe, painless, and non-invasive brain stimulation technique. In tACS, a weak (≤ 4 mA) sinusoidal electrical current is applied to the scalp through two or more surface electrodes (Antal et al., 2008). *In vivo* evidence from animal models has shown that tACS can entrain endogenous neural oscillations to the frequency and phase of the exogenous electric current (for a review of tACS mechanisms, see Wischnewski et al., 2023). If beta tACS can entrain sensorimotor beta oscillations in the human brain, it may be able to improve the overall efficiency of inhibitory and excitatory circuits (Fries, 2005, 2015; Uhlhaas et al., 2009). As a result, tACS might lead to more efficient ERD and ERS, which could be functionally beneficial for voluntary movement (Hervault et al., 2021). Several studies have investigated the effect of theta and alpha tACS on neural oscillations, observing significant frequency-dependent increases in magnetoencephalography/electroencephalography (M/EEG) power post-stimulation (e.g., D’Atri et al., 2019; Neuling et al., 2013; Zaehle et al., 2010), lasting for up to 70 min (Kasten et al., 2016). These increases in M/EEG power suggest that tACS can entrain neural oscillations in humans, and that this effect can outlast the stimulation period, likely through synaptic plasticity-like mechanisms (Vossen et al., 2015; Wischnewski et al., 2019). However, the effect of beta tACS on sensorimotor beta oscillations remains unclear.

Some studies have shown that beta tACS (20 Hz) can induce a significant increase in resting-state beta oscillations and event-related beta oscillations (Battaglini et al., 2020; Lyzhko et al., 2023; Nakazono et al., 2020; Suzuki et al., 2022), while other studies have not observed significant changes (Akaiwa et al., 2024; Harada et al., 2020; Lafleur et al., 2021; Rumpf et al., 2019; Sugata et al., 2018). It is unclear why results have been inconsistent, although, these conflicting results might be explained by differences in stimulation parameters between studies (e.g., stimulation intensity, electrode montage, and stimulation duration). Of these tACS parameters, the effect of stimulation intensity (all reported as peak-to-peak values throughout this article) on sensorimotor beta power remains to be systematically investigated. Dynamic systems theory (Pikovsky et al., 2001) suggests that higher intensities of tACS would induce greater entrainment. *In vivo* animal models support this notion, demonstrating a linear intensity-response relationship for neural spike timing (Asan et al., 2020; Huang et al., 2021; Johnson et al., 2020; Krause et al., 2022). While no human studies have directly examined the effect of tACS intensity on entrainment, Moliadze et al. (2012) found an intensity-response relationship in human corticospinal excitability. Gamma tACS (140 Hz) positioned over the left primary motor cortex (left M1) at 0.4 mA reduced corticospinal excitability, at 0.6–0.8 mA had no effect on corticospinal excitability, and at 1.0 mA induced an excitatory effect on corticospinal excitability. However, 2.0 mA stimulation did not induce greater increases in corticospinal excitability than 1.0 mA (Shorafa et al., 2021). Interestingly, Wang Y. et al. (2022) found that the intensity-response relationship in human neural oscillations depended on participant brain states: 2.0 mA alpha tACS induced greater increases in resting-state alpha power compared to 1.0 mA tACS when participants had their eyes open, but not when they had their eyes closed. When testing higher stimulation intensities, De Koninck et al. (2021) found that 1.0 mA alpha tACS induced a significantly greater increase in posterior alpha power, compared to 4.0 to 6.0 mA stimulation. Together, these findings suggest a complex relationship between the stimulation intensity and the neurophysiological response to tACS in humans.

In the current study, we examined the effect of different intensities of beta tACS (20 Hz) on sensorimotor oscillations in healthy young adult humans. Our primary aim was to investigate intensity-dependent changes in resting-state beta oscillations, for which we selected 20 Hz as it is the most commonly used frequency in beta tACS literature, enabling direct comparison with previous studies. As a secondary aim, we examined how different intensities of beta tACS affect movement-related oscillations, which can be partially addressed with 20 Hz stimulation, although we acknowledge that this stimulation frequency sits at the boundary of low and high beta. Event-related EEG recordings captured movement-related beta activity elicited by participants performing self-paced voluntary hand movements. High-definition tACS was delivered with the center electrode positioned over the hand-area of left M1 at varying intensities (0.5 mA, 1.0 mA, 1.5 mA, peak-to-peak), and sham stimulation was included as a control. We selected these stimulation intensities to systematically investigate effects below and above the most commonly used intensity of 1.0 mA, based on evidence suggesting potential inhibitory effects at lower intensities and facilitatory effects at higher intensities (e.g., Moliadze et al., 2012; Shorafa et al., 2021). Both resting-state and event-related sensorimotor oscillations were measured using EEG power. Based on dynamic systems theory and findings from the evaluation of tACS intensity within *in vivo* animal models, it was hypothesized that beta tACS would linearly increase resting-state and event-related beta power in the region of interest (ROI) centered around left M1. To further understand the frequency-specificity of beta tACS, we examined power changes within three other frequency bands: theta, alpha, and gamma. Additionally, we assessed the region-specificity of beta tACS by comparing the changes at the stimulated ROI (over left M1) to the changes at a non-stimulated contralateral ROI (over right M1). We also explored whether tACS modulated participants’ peak beta frequency, and whether the change in event-related power following tACS was associated with the difference between participants’ endogenous peak beta frequency and the exogenous stimulation frequency.

## 2 Materials and methods

The participants and experimental procedures in this study were identical to those reported in Wansbrough et al. (2024a), which comprehensively investigated the effect of beta tACS positioned over left M1 on M1-M1 connectivity. The current study presents a distinct analysis of the same dataset, exploring the effect of beta tACS on sensorimotor power to address complementary research questions.

### 2.1 Participants

Forty-seven healthy young adults were originally recruited to attend four experimental sessions. Of those, 24 participants did not complete all four sessions: 4 attended three sessions, 10 attended two sessions, and 10 attended one session before dropping out due to unforeseen personal circumstances (*n* = 14), COVID-19 lockdown (*n* = 8), or minor adverse events (1 experienced a headache and 1 experienced dizziness). A total of 23 healthy young adults participated in all four experimental sessions (13 female; age range = 18 to 34 years; mean age = 24.30 ± 4.84 years). Participants were right-handed, as assessed by the Edinburgh Handedness Inventory (Oldfield, 1971; range = 41.18–100.00; M = 87.59, SD = 16.22), had no contraindications to non-invasive brain stimulation (Rossi et al., 2009; Rossini et al., 1994), and had no history of neurological conditions. All participants provided written informed consent. The experiment was conducted in accordance with the Declaration of Helsinki and was approved by the Murdoch University Human Research Ethics Committee (2018/098).

### 2.2 Experimental design

The experiment was a sham controlled, triple-blinded, within-subjects design. Each participant completed four sessions, separated by at least 72 h (mean inter-session interval = 11.68 ± 6.46 days; Chaieb et al., 2014; Saito et al., 2022; Stecher et al., 2017). The independent variable was stimulation intensity: sham, 0.5 mA, 1.0 mA, and 1.5 mA (peak-to-peak). The order of stimulation intensities was counterbalanced across participants. Individual participants were tested at the same time of day so that inter-session differences in post-tACS power could not be attributed to the time of testing (Wilson et al., 2014). Participants were not informed of the intensities applied in each session, being only informed that variations in stimulation settings were being investigated, including a sham session. At the end of the fourth session, it was revealed that each session varied in stimulation intensity. The researcher who conducted data collection and analysis (KW) was blinded to the stimulation intensities. An independent researcher (AMV) pre-set the stimulation parameters and randomly assigned each intensity to a label: “A,” “B,” “C,” or “D.” The researcher conducting the analysis (KW) remained blinded to the conditions until all analyses were completed.

### 2.3 Transcranial alternating current stimulation

High-definition tACS (HD-tACS) was delivered through conductive round rubber electrodes (2 cm diameter; 3.14 cm^2^ area) via a neuroConn DC-STIMULATOR MC (NeuroConn, Ilmenau, Germany). To reduce impedance, a Ten20 conductive paste was placed between the surface of the electrodes and the scalp. Impedance was kept below 50 kΩ.

A 4 × 1 HD-tACS electrode montage was used, as it has been shown to deliver a more focal current to than the standard bipolar tACS montage (Datta et al., 2008; Edwards et al., 2013). The center electrode was placed over the left M1 representation of the first-dorsal interosseous (FDI), which was located using transcranial magnetic stimulation (TMS; full details regarding TMS procedure provided in Supplementary Methods). Current densities for the center electrode were 0.159 mA/cm^2^ for 0.5 mA stimulation, 0.318 mA/cm^2^ for 1.0 mA stimulation, and 0.478 mA/cm^2^ for 1.5 mA stimulation. The four return electrodes were placed at a 50 mm radius from the center electrode. Placement of the return electrodes was based on electric current simulations in a model of the average adult head (MNI152; conducted with SimNIBS v3.2.0; Thielscher et al., 2015). Current densities for each of the return electrodes were 0.040 mA/cm^2^ for 0.5 mA stimulation, 0.080 mA/cm^2^ for 1.0 mA stimulation, and 0.120 mA/cm^2^ for 1.5 mA stimulation. Electric field models can be found in the Supplementary Methods.

Sinusoidal stimulation was delivered at 20 Hz with zero DC-offset, for 20 min. Both this stimulation frequency and duration have been widely used in previous tACS studies, facilitating comparisons with existing literature. Notably, both parameters have been shown to induce changes in neurophysiological measures (e.g., resting-state EEG, corticospinal excitability and functional near-infrared spectroscopy; Berger et al., 2018; Heise et al., 2016; Neuling et al., 2013) and behavior (e.g., motor learning, Pollok et al., 2015). For all real stimulations, there was a 30 s ramp up period to the target intensity and a 30 s ramp down period (Woods et al., 2016). For the sham stimulation, a 30 s ramp up was immediately followed by a 30 s ramp down at 0 and 20 min. For all participants, the sham tACS current ramped up to a peak-to-peak intensity of 1.5 mA (the highest stimulation intensity that was investigated in this experiment). Results from previous studies indicated that this sham tACS protocol was sufficient for eliciting the typical sensation usually perceived at the onset of active tACS (Ambrus et al., 2012; Gandiga et al., 2006; Woods et al., 2016).

### 2.4 Electroencephalography

EEG was collected with a 128-electrode EGI HydroCel™ Geodesic Sensor Net (Electrical Geodesics, Inc., Eugene, OR), following the international 10–20 system of electrode placement (Klem et al., 1999; Jurcak et al., 2007). EEG signals were acquired using EGI Net Amps 300 amplifiers and Netstation 4.5.6, band pass filtered (0.05 to 100 Hz), and digitized at a sampling rate of 1,000 Hz. Signals were referenced to Cz during recording, and impedance was kept below 50 kΩ (Nelson et al., 2017; Alhajri et al., 2018; Angelini et al., 2018; Tatti et al., 2019) as per the manufacturer’s recommendation (Magstim EGI, Eugene, OR). The HydroCel Geodesic Sensor Net allowed us to place the tACS electrodes without having to remove the EEG net.

#### 2.4.1 EEG recording procedure

Two types of EEG recordings were taken: (1) resting-state recordings; (2) event-related recordings. For the resting-state recordings, participants were instructed to keep their eyes open and look straight ahead at a fixation cross for 3 min. For the event-related recordings, participants were instructed to perform self-paced isometric flexions of the right index finger at approximately 10 s intervals. An index finger flexion was chosen, as it has been shown to elicit the three event-related changes in beta activity over the sensorimotor cortex (e.g., Rimbert et al., 2018). Movements were self-paced to engage the neural processes involved in generating internally motivated voluntary movements, which slightly differ from externally motivated movements (e.g., reduced motor preparation; Brass and Haggard, 2008; Haggard et al., 2006). The tip of the index finger was placed on an 8 mm 10 N/2.2 lb. SingleTact force sensor (SingleTact, Glasgow, UK) that was permanently secured on a computer mouse. Participants performed a total of 60 index finger flexions, which were split into two blocks of 30 flexions. Before the experimental session began, participants completed one 60 s finger flexion training block with a go signal (“press” presented on a screen every 10 s) and one block that was self-paced, with verbal feedback from the experimenter. For the duration of event-related recordings, participants were instructed to look straight ahead at a fixation cross to avoid random eye movements, and minimize any other movement (e.g., blinking, swallowing, etc.).

#### 2.4.2 Force sensor event trigger

As stated above, the event-related recordings required participants to press into a force sensor. This was done so that the onset of movement could be registered as a NetStation (Electrical Geodesics, Inc.) digital input event for data analysis. The force sensor output was digitized at a sampling value of 5,000 Hz (CED Power1401), data were acquired using Signal (version 6.02, Cambridge Electronic Design), and event-triggers were generated in NetStation at the onset of movement, using a custom-written Signal sequencer. The onset of movement was defined as the moment at which the force-trace exceeded a pre-determined threshold (which was determined for each participant during practice trials).

### 2.5 Procedure

**Figure 1:**
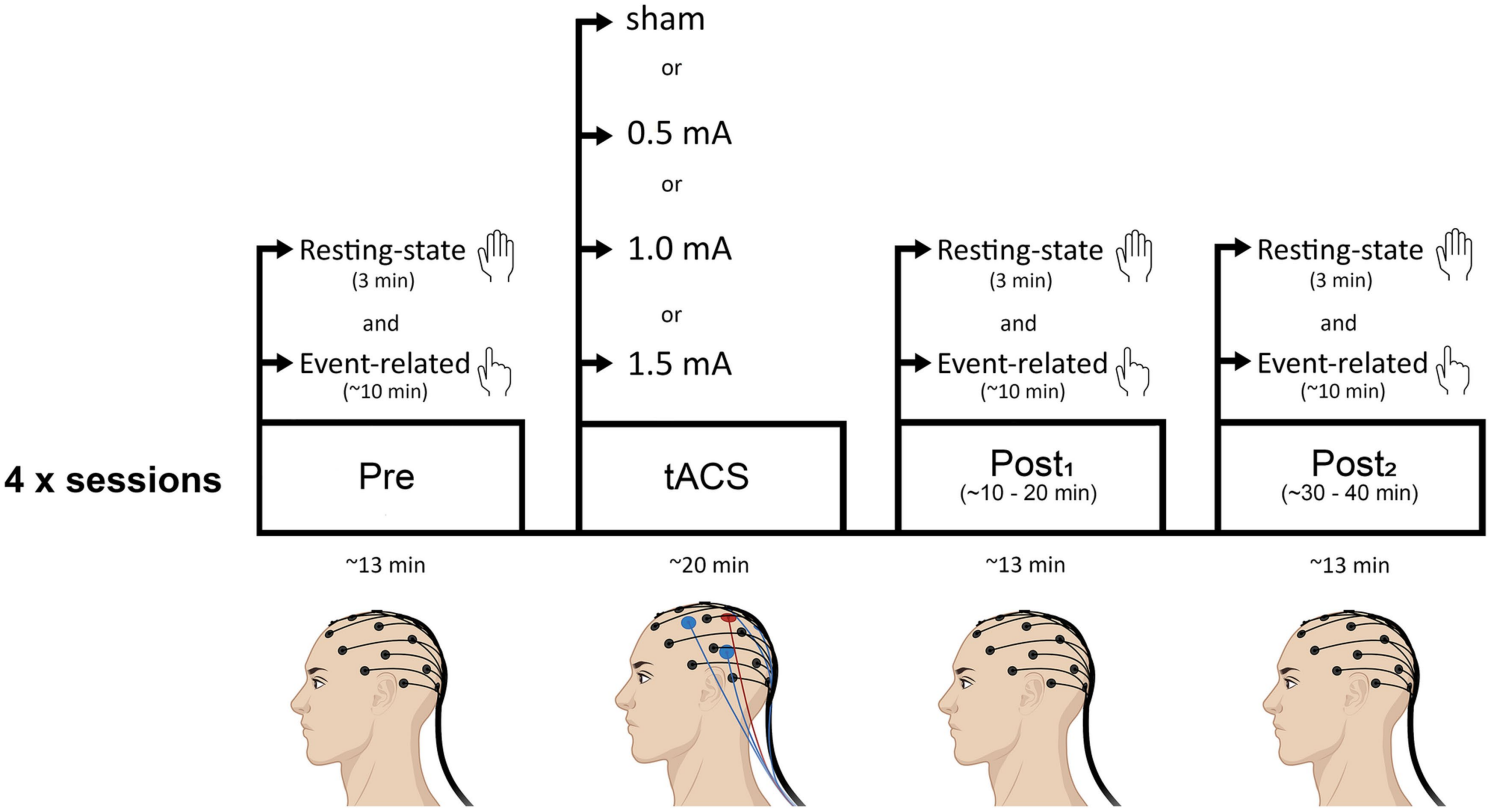
Schematic of the experimental timeline. Participants attended four sessions (each separated by a minimum of 72 h): three real tACS sessions in which the intensity of stimulation was varied (0.5 mA, 1.0 mA, or 1.5 mA, peak-to-peak), and a sham (control) stimulation session. Sessions were counterbalanced across participants. Beta tACS was delivered with the center electrode positioned over the hand-area of left M1 at 20 Hz for 20 min. Resting-state EEG and event-related EEG recordings were obtained before tACS (pre), and at two time points following tACS (post_1_, post_2_). Resting-state EEG recordings always preceded event-related EEG recordings.

Figure 1 shows a schematic of the experimental timeline. At the beginning of each session, the left M1 representation of the FDI was located for tACS electrode placement and the EEG cap was fitted. After this, baseline measures of resting-state and event-related EEG were recorded. Then, tACS electrodes were placed underneath the EEG net, onto the scalp, and either real or sham stimulation was delivered for 20 min. EEG was not recorded during the stimulation period. During the 20 min tACS stimulation, participants were seated comfortably in a chair and instructed to keep their eyes open, looking straight ahead. The researcher sat next to the participant and engaged in light conversation to maintain alertness without excessive cognitive stimulation. Participants were asked to remain relaxed and minimize movements throughout the stimulation period. Following stimulation, the tACS electrodes were removed and both resting-state and event-related EEG were recorded (as per baseline measures) at two time-points: ~5 min following tACS (post_1_; range = 3 to 8 min post-tACS; mean time = 5.5 min post-tACS) and ~25 min following tACS (post_2_; range = 20 to 29.5 min post-tACS; mean time = 24.6 min post-tACS). The purpose of the second recording was to determine whether any changes induced by tACS persisted beyond the immediate post-stimulation period, providing insight into the longevity of the stimulation effects. Before each EEG recording block, experimenters ensured that impedance levels were below threshold. The delay between stimulation end and the first post-tACS recording was due to the time required to remove the tACS electrodes and bring impedance below threshold. Each session lasted approximately 2.5 h.

### 2.6 Tolerability and blinding

At the end of each session, participants completed a 12-item self-report questionnaire regarding perceived sensations and adverse effects induced by tACS (Fujiyama et al., 2023). The questionnaire evaluated specific sensations commonly associated with transcranial electrical stimulation (itching, tingling, burning, pain, warmth/heat, metallic taste, fatigue, headache, etc.) using a 5-point scale (0 = nothing, 4 = very strong). Participants also indicated when sensations occurred (start, middle, or end of stimulation). Additionally, to monitor blinding effectiveness, participants were asked whether they thought they received real or sham tACS and to rate their confidence in their answer on a scale of 1 (not confident at all) to 10 (very confident).

### 2.7 Data analysis

#### 2.7.1 EEG pre-processing

EEG data were pre-processed using the EEGLAB toolbox (Delorme and Makeig, 2004) through the MATLAB environment (MathWorks, R2020b). All EEG data were down-sampled to 500 Hz, bandpass filtered from 0.5 to 95 Hz, and notch filtered at 50 Hz. The data were then epoched: resting-state data were divided into 2,000 ms segments; event-related data were segmented from −2,500 to 4,500 ms relative to stimulus onset. Bad channels and noisy epochs were then visually identified and manually removed, and all removed channels were interpolated. The data were then re-referenced to the average, using the *fullRankAveRef* EEGLAB plugin. Next, independent component analysis (ICA) was performed, using the Infomax algorithm. Following ICA, components containing artifacts clearly distinguished from brain-driven EEG signals (e.g., ocular, vascular, and myogenic artifacts) were visually identified and subtracted from the data. During EEG pre-processing, two resting-state data sets and three event-related data sets (across 4 participants) were identified as having a large number of artifact contaminated epochs. These data sets contained <11 useable trials, below the recommended minimum of 20 trials (Cohen, 2014). Participants with an insufficient number of trials were excluded from further analysis, resulting in sample sizes of *N* = 21 for the resting-state analyses and *N* = 20 for the event-related analyses.

#### 2.7.2 Computing EEG power

Using custom MATLAB scripts, power values were computed from the pre-processed resting-state data and the pre-processed event-related data. Power values were calculated for the left M1 ROI (tACS target) and the right M1 ROI. The right M1 ROI was analyzed to explore whether tACS effects extended to this functionally connected region. While electrodes C3 and C4 in international 10–20 EEG system correspond to the approximate position of the left M1 and right M1, respectively (Jurcak et al., 2007), we analyzed the activity of electrode clusters—a cluster of seven electrodes centered at C3 (C3 cluster: C3, FC3, C1, FC5, CP1, C5, CP3), and a cluster of seven electrodes centered at C4 (C4 cluster: CP2, CP4, C6, C2, FC6, FC4).

##### 2.7.2.1 Computing resting-state power

Power values were initially computed for each electrode within the C3 and C4 clusters. The time series of each electrode was convolved with complex Morlet wavelets for frequencies between 4 and 90 Hz, in 1 Hz increments (87 wavelet frequencies in total). The length of the wavelets started at 3 cycles for the lowest frequency, and logarithmically increased as the frequencies increased, such that the length was 13 cycles for the highest frequency. This approach provided a balance between temporal and frequency precision (Cohen, 2014). Power values were obtained by multiplying the resultant analytic signal by its complex conjugate. To minimize the effects of edge artifacts, power values were only obtained from time windows of 400–1,600 ms (at 20 ms intervals) within each 2,000 ms epoch. Then, an average power value was calculated for each electrode cluster at each time-point, frequency, and trial. Finally, the power values were converted to dB.

##### 2.7.2.2 Computing event-related power

**Figure 2:**
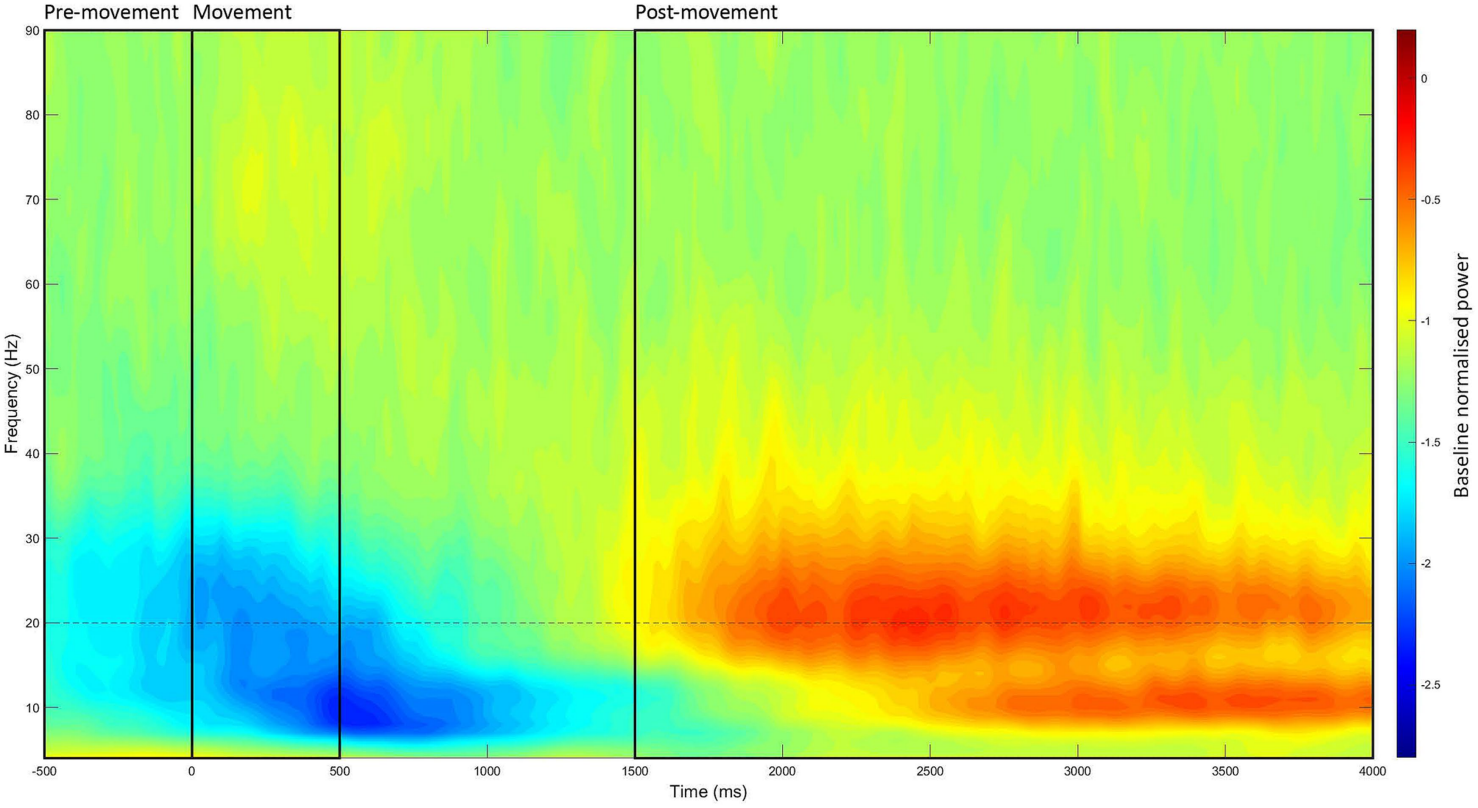
Grand-averaged event-related time-frequency power at the left M1 ROI. Time-frequency power estimates were averaged across all recording blocks, participants, and stimulation intensities, then baselined to the period of −2,000 to −1,000 ms. Through visual inspection, the three movement periods were identified (marked in black boxes): (1) the pre-movement period (−500 to 0 ms), (2) the movement period (0–500 ms), and (3) the post-movement period (1,500–4,000 ms).

This process was identical to the resting state, with three exceptions. First, the time window of interest was −500 to 4,000 ms (at 20 ms intervals) relative to stimulus onset. Second, the power values were baselined to the period of −2,000 to −1,000 ms. Third, power estimates were separated into three different movement periods within each −500 to 4,000 ms window: (1) pre-movement period (−500 to 0 ms), (2) movement period (0–500 ms), and (3) post-movement period (1,500–4,000 ms). These periods were analyzed separately as each reflects a different aspect of movement, which are underpinned by slightly different mechanisms (Kilavik et al., 2013). The time-window of each movement period was determined through visual inspection of the grand-averaged data (see Figure 2).

#### 2.7.3 Statistical analyses

In this study, the data were analyzed with generalized linear mixed models (GLMMs) instead of ANOVAs, due to mixed models accounting for inter-individual variability, as inter-individual response variability is a known issue in NIBS (for a review, see Guerra et al., 2020). Statistical analyses and visualization of the results were performed via customized scripts in MATLAB, and the software package R for Statistical Computing version 2023.09.0 + 463 (R Core Team, 2023), using packages “tidyverse” (Wickham, 2023), “DescTools” (Signorell, 2023), “janitor” (Firke, 2023), “car” (Fox et al., 2023), and ggplot2 (Wickham et al., 2023). The “lme4” package (Bates et al., 2023) was used to construct GLMMs, fitted by means of the glmer() function. For *post-hoc* analyses, we used the “emmeans” package (Lenth, 2023) to perform pairwise comparisons between estimated marginal means derived from our GLMM models. These comparisons were executed using the contrast() function with Bonferroni correction to account for multiple comparisons. To quantify the magnitude of differences, effect sizes were calculated using the eff_size() function, providing standardized measures of the differences between means.

##### 2.7.3.1 Analyzing control measures

In addition to reporting the presence and strength of any tACS-related sensations, participants completed a questionnaire assessing their sleep quality and quantity (for the night before the session), as well as the amount of caffeine and alcohol consumed in the 12 h prior to each experimental session. Separate GLMMs were conducted to examine differences between stimulation intensities in the perceived strength of tACS sensations, as well as differences in pre-session sleep quality, sleep quantity, caffeine intake, and alcohol intake. All models included participant-specific random intercepts. Model estimates were obtained for the fixed effect of INTENSITY (sham, 0.5 mA, 1.0 mA, and 1.5 mA). Akaike’s Information Criterion (AIC) values were used to determine the best distribution and function for each model (Burnham and Anderson, 2004).

##### 2.7.3.2 Analyzing changes in resting-state and event-related power

Trial-level estimates of resting-state power were obtained by averaging power values across the time points within each epoch. Trial-level estimates of event-related power were obtained by averaging power values across the time points within each movement period, within each epoch. Both resting-state and event-related power values were averaged across five frequency bands: theta (4–7 Hz), alpha (8–12 Hz), beta (13–30 Hz), and gamma (60–90 Hz).

The effects of tACS on resting-state power and event-related power were investigated with separate GLMMs, fitted with a Gaussian distribution and log function. These models were determined as having the best fit, based on AIC values. For the event-related data, separate analyses were performed for each of the three movement periods (pre-movement, movement, post-movement). All models included participant-specific random intercepts to account for inter-individual variability in the data. Model estimates were obtained for the fixed effects of INTENSITY (sham, 0.5 mA, 1.0 mA, and 1.5 mA), TIME (pre, post_1_, and post_2_), FREQUENCY (theta, alpha, beta, and gamma), and REGION (C3 cluster and C4 cluster).

##### 2.7.3.3 Analyzing event-related peak beta

We also explored whether tACS modulated participants’ peak beta frequencies, relative to the stimulation frequency of 20 Hz. Within each movement period, we extracted each participants’ peak beta frequency for each trial, defined as the beta frequency with the greatest ERD/ERS in the corresponding time-frequency window. Trial-level estimates of the difference between peak beta frequency and tACS frequency were obtained by subtracting 20 Hz from the peak beta frequency. This difference will be referred to as the endogenous-exogenous frequency difference. The effect of tACS was investigated with separate GLMMs for each movement period. All models included participant-specific random intercepts. Model estimates were obtained for the fixed effects of INTENSITY, TIME, and REGION. For all GLMM analyses, statistical significance was set at α = 0.05, and significant effects were investigated with custom Bonferroni-corrected contrasts.

We further explored whether the pre-tACS endogenous-exogenous frequency difference was associated with the post-tACS changes in event-related power. This was investigated with cluster-based permutation tests using Spearman’s *ρ*. To overcome the multiple-comparisons problem, cluster-based permutation statistics are most appropriate for exploratory analyses in time-frequency data (Cohen, 2014). For each movement period, region, and stimulation intensity, we extracted participants’ trial-averaged: (1) pre-tACS endogenous-exogenous frequency difference; (2) percent-change in event-related power (*Δ* event-related power) between time points (i.e., between measurement blocks). Separate tests were conducted to examine associations with Δ event-related power between each time point (i.e., Δ pre to post_1_, Δ pre to post_2_, and Δ post_1_ to post_2_). For each test, sample points with values exceeding α = 0.05 were clustered according to spectral-temporal adjacency, with separate clusters for positive and negative values. The size of each cluster was determined by summing the absolute statistical values within it. The largest cluster size of each iteration was selected to form the permutation distribution. Clusters from the real data were compared to this permutation distribution, and cluster sizes that exceeded the 97.5th percentile of this distribution were considered significant.

## 3 Results

Stimulation intensity-related changes in power over time were of primary interest in the present study. As such, all the main effects and only the highest level of interaction involving both INTENSITY and TIME as factors will be described in detail.

### 3.1 Control measures

As reported in our previous work, analyses of sensations and blinding efficacy revealed some trends toward participants being able to distinguish higher intensity stimulation from sham, though confidence ratings did not differ between conditions. For detailed analyses of these control measures, readers are referred to Wansbrough et al. (2024a). Detailed ratings of participants’ sensations experienced for each stimulation intensity are provided in the Supplementary Results. The results presented below should be interpreted with consideration of these blinding limitations.

### 3.2 Changes in resting-state power

The GLMM analysis of resting-state power found significant main effects for all four factors: INTENSITY (sham, 0.5 mA, 1.0 mA, 1.5 mA), TIME (pre, post_1_, post_2_), FREQUENCY (theta, alpha, beta, gamma), and REGION (C3 cluster, C4 cluster; all *χ*^2^s ≥ 221.36, all *p*s < 0.001). Importantly, these effects were qualified by a significant four-way INTENSITY × TIME × FREQUENCY × REGION interaction (*χ*^2^ (18, *N* = 21) = 70.20, *p* < 0.001). All lower-order interactions contained within this four-way interaction were also significant (all *χ*^2^s ≥ 103.02, all *p*s < 0.001).

To facilitate interpretation of the significant four-way interaction, *post-hoc* analyses were conducted separately for each frequency band. *Post-hoc* analyses within each frequency band comprised four sets of pairwise comparisons. As the primary interest of the study was to examine the effect of stimulation intensity on resting-state power, the first set of comparisons aimed to determine which stimulation intensities showed significant power changes at the C3 cluster over time. To determine whether the changes were tACS-related, the second set of pairwise comparisons examined whether any of the real stimulation intensities showed significantly different changes in power compared to sham stimulation. If multiple real stimulation intensities showed significant differences relative to sham, a third set of comparisons was performed on the real stimulation intensities to examine whether the change in power significantly differed between these stimulation intensities. The fourth set of pairwise comparisons was then performed to determine whether changes in resting-state power were local to the stimulation site (C3 cluster), by contrasting these changes to those in a contralateral electrode cluster (C4 cluster). It is important to note that there were significant differences in power between some of the stimulation intensities at baseline. Tables of these results can be found in the Supplementary Results. For this reason, comparisons between stimulation intensities were only performed on the relative change between two time points. It is possible that baseline differences may have affected the capacity for change, so comparisons between stimulation intensities should be interpreted with caution.

#### 3.2.1 Beta tACS induced little change in resting-state beta power at the left M1 ROI

**Figure 3:**
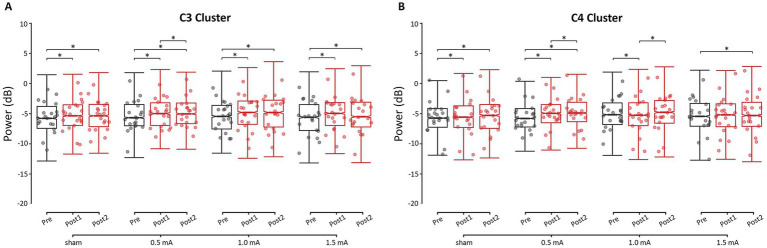
Changes in resting-state beta power for each time-point and stimulation intensity, for **(A)** the C3 cluster (i.e., left M1 ROI) and **(B)** the C4 cluster (i.e., right M1 ROI). * = significant change between time-points at α = 0.05. Data points reflect participant averages. The height of the notches reflects the median ± 1.57 × IQR/sqrt(n) where IQR is the interquartile range defined by the 25th and 75th percentiles and n is the number of data points.

Overall, the theta, alpha, and beta frequency bands showed a similar post-tACS pattern of change across stimulation intensities, while responses in gamma power differed. Here, we focus on describing the *post-hoc* analyses of resting-state power in the beta band, as this was the target frequency. Detailed results for each of the other frequency bands can be found in the Supplementary Results. As shown in the left panel of Figure 3, all four stimulation intensities showed increased beta power at the C3 cluster from pre to post_1_ (|*z*s| ≥ 3.578, *p*s ≤ 0.001, |*d*s| ≥ 0.008) and from pre to post_2_ (|*z*s| ≥ 4.267, *p*s < 0.001, |*d*s| ≥ 0.010). Most stimulation intensities did not show a significant change from post_1_ to post_2_ (|*z*s| ≤ 0.951, *p*s ≥ 0.608, |*d*s| ≤ 0.002), with only 0.5 mA stimulation showing a significant change: a further increase in beta power (*z* = −2.689, *p* = 0.022, *d* = −0.006). These increases in power at C3 were unlikely due to tACS, as the extent of changes following real stimulation was not greater than the changes following sham stimulation (|*z*s| ≤ 1.493, *p*s = 1.000, |*d*s| ≤ 0.029).

#### 3.2.2 Region-specific changes in resting-state beta power were observed following 1.0 mA and 1.5 mA stimulation

Region-specific changes in beta power were observed following 1.0 mA and 1.5 mA stimulation (|*z*s| ≥ 2.652, *p*s ≤ 0.024, |*d*s| ≥ 0.050), but not 0.5 mA or sham stimulation (|*z*s| ≤ 2.064, *p*s ≥ 0.117, |*d*s| ≤ 0.041). Following 1.0 mA stimulation, from pre to post_1_, beta power increased at C3 (*z* = −4.713, *p* < 0.001, *d* = −0.011) and decreased at C4 (*z* = 3.014, *p* = 0.008, *d* = 0.007). At post_2_, C3 beta power remained elevated relative to baseline (*z* = −4.267, *p* < 0.001, *d* = −0.010), while C4 beta power shifted back toward baseline levels (*z* = −3.579, *p* = 0.001, *d* = −0.008). In contrast, 1.5 mA stimulation increased C3 beta power from pre to post_1_ (*z* = −6.384, *p* < 0.001, *d* = −0.015) without affecting C4 (*z* = −1.670, *p* = 0.285, *d* = −0.004). By post_2_, 1.5 mA stimulation showed delayed increases in beta power at both C3 (*z* = −5.411, *p* < 0.001, *d* = −0.013) and C4 (*z* = −2.783, *p* < 0.016, *d* = −0.007).

### 3.3 Changes in event-related power

#### 3.3.1 Beta tACS induced bilateral intensity-dependent changes in pre-movement (−500 to 0 ms) broadband (4–90 Hz) power

The GLMM analysis of pre-movement power found significant main effects for INTENSITY and FREQUENCY (*χ*^2^s ≥ 13.626, *p*s ≤ 0.004), but not TIME or REGION (*χ*^2^s ≤ 1.142, *p*s ≥ 0.565). The analysis also revealed a significant two-way interaction between INTENSITY and TIME (*χ*^2^ = 19.434, *p* = 0.004). All other interactions were non-significant (*χ*^2^s ≤ 17.640, *p*s ≥ 0.479). These results indicate that tACS induced stimulation intensity-specific changes in pre-movement power. However, these changes were not frequency-specific, indicating that beta tACS did not selectively modulate the pre-movement beta ERD. Additionally, these changes were not region-specific, indicating that tACS exerted similar effects bilaterally, across the stimulated C3 cluster (left M1 ROI) and the unstimulated C4 cluster (right M1 ROI). The highest level of interaction—the two-way INTENSITY × TIME interaction—was further investigated with *post-hoc* comparisons.

**Figure 4:**
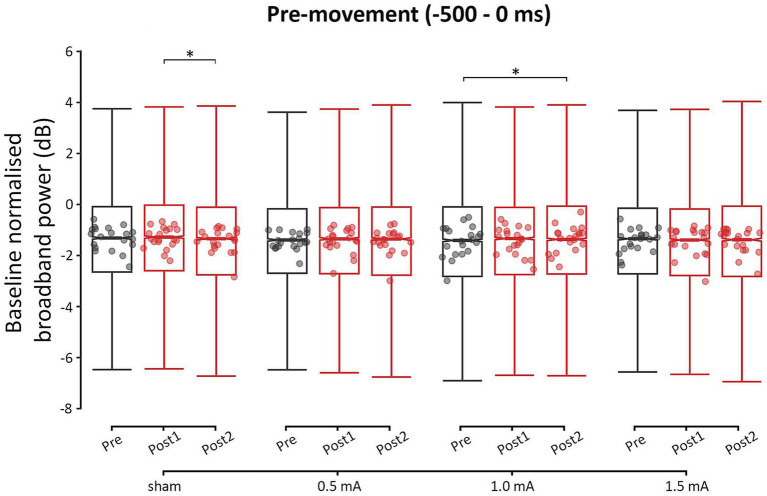
Event-related changes bilateral broadband power (4 to 90 Hz) of the pre-movement period (−500 to 0 ms), for each stimulation intensity. Power values have been baseline-normalized to the period of −2,000 to −1,000 ms. * = significant change between time-points at α = 0.05. Data points reflect participant averages. The height of the notches reflects the median ± 1.57 × IQR/sqrt(n) where IQR is the interquartile range defined by the 25th and 75th percentiles and n is the number of data points.

Figure 4 shows the pre-movement power values for each time-point and stimulation intensity. It is important to note that there were significant differences in power between some of the stimulation intensities at baseline. Tables of these results can be found in the Supplementary Results. For this reason, comparisons between stimulation intensities were only performed on the relative change between two time points, though these comparisons should be interpreted with caution. From pre to post_1_, none of the stimulation intensities showed a significant change in pre-movement power (|*z*s| ≤ 1.360, *p*s ≥ 0.362, |*d*s| ≤ 0.001). From pre to post_2_, there was a significant increase in pre-movement power only following 1.0 mA stimulation (|*z*| = −3.045, *p* = 0.007, |*d*| = −0.003). This increase was likely due to tACS, as there were no changes following sham stimulation from pre to post_2_ (*z* = 1.917, *p* = 0.134, *d* = 0.002). From post_1_ to post_2_, the sham condition showed a significant decrease in power (*z* = 2.961, *p* = 0.009, *d* = 0.003), while the real stimulation intensities showed no significant changes in power (|*z*s| ≤ 1.697, *p*s ≥ 0.206, |*d*s| ≤ 0.002). It is possible that the real stimulation intensities promoted stability in pre-movement power, or that the decrease following sham stimulation was a chance occurrence.

#### 3.3.2 Beta tACS induced intensity-dependent changes in bilateral theta and alpha power, but not beta or gamma power, during movement (0–500 ms)

The GLMM analysis of movement power found significant main effects for all four factors: INTENSITY, TIME, FREQUENCY, and REGION (all χ^2^s ≥ 10.46, all *p*s ≤ 0.005). The analysis also revealed a significant three-way interaction of INTENSITY, TIME, and FREQUENCY (χ^2^ (18, *N* = 20) = 30.83, *p* = 0.030). All other interaction effects were non-significant (χ^2^s ≤ 16.34, *p*s ≥ 0.060). These results indicate that tACS induced frequency-specific and intensity-specific, but not region-specific, changes in movement power. The highest level of interaction—the three-way INTENSITY × TIME × FREQUENCY interaction—was further investigated with *post-hoc* comparisons.

**Figure 5:**
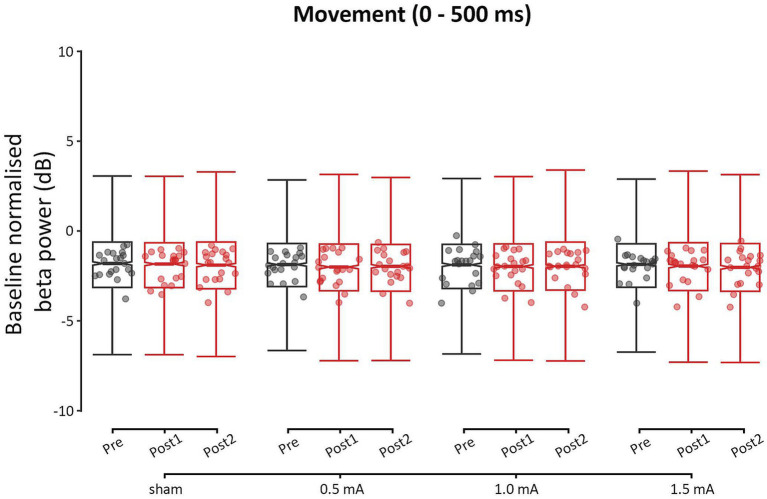
Event-related changes bilateral beta power (13–30 Hz) of the movement period (0–500 ms), for each stimulation intensity. Power values have been baseline-normalized to the period of −2,000 to −1,000 ms. Data points reflect participant averages. The height of the notches reflects the median ± 1.57 × IQR/sqrt(n) where IQR is the interquartile range defined by the 25th and 75th percentiles and n is the number of data points.

Overall, none of the real stimulation intensities induced a change in beta or gamma power, relative to sham stimulation (|*z*s| ≤ 2.668, *p*s ≥ 0.138, |*d*s| ≤ 0.238). The lack of change in beta power indicates that the movement beta ERD was not modulated by 20 Hz tACS (see Figure 5). In contrast, intensity-dependent changes in theta and alpha power were observed, indicating that cross-frequency neuromodulation occurred. Detailed results for the theta, alpha, and gamma frequencies can be found in the Supplementary Results.

#### 3.3.3 Beta tACS induced bilateral intensity-dependent changes in post-movement (1,500–4,000 ms) broadband (4–90 Hz) power

The GLMM analysis of post-movement power revealed significant main effects for all four factors: INTENSITY, TIME, FREQUENCY, and REGION (all *χ*^2^s ≥ 11.43, all *p*s ≤ 0.010) and a significant two-way INTENSITY × TIME interaction (χ^2^ (6, *N* = 20) = 34.51, *p* < 0.001), with all higher-order interactions non-significant (χ^2^s ≤ 20.05, *p*s ≥ 0.330). Similar to the pre-movement period, tACS induced stimulation intensity-specific changes in post-movement power, however, these changes were not frequency-specific or region-specific. As these changes were not frequency-specific, these results indicate that beta tACS did not selectively modulate the post-movement beta ERS. The highest level of interaction—the two-way INTENSITY × TIME interaction—was further investigated with *post-hoc* comparisons.

**Figure 6:**
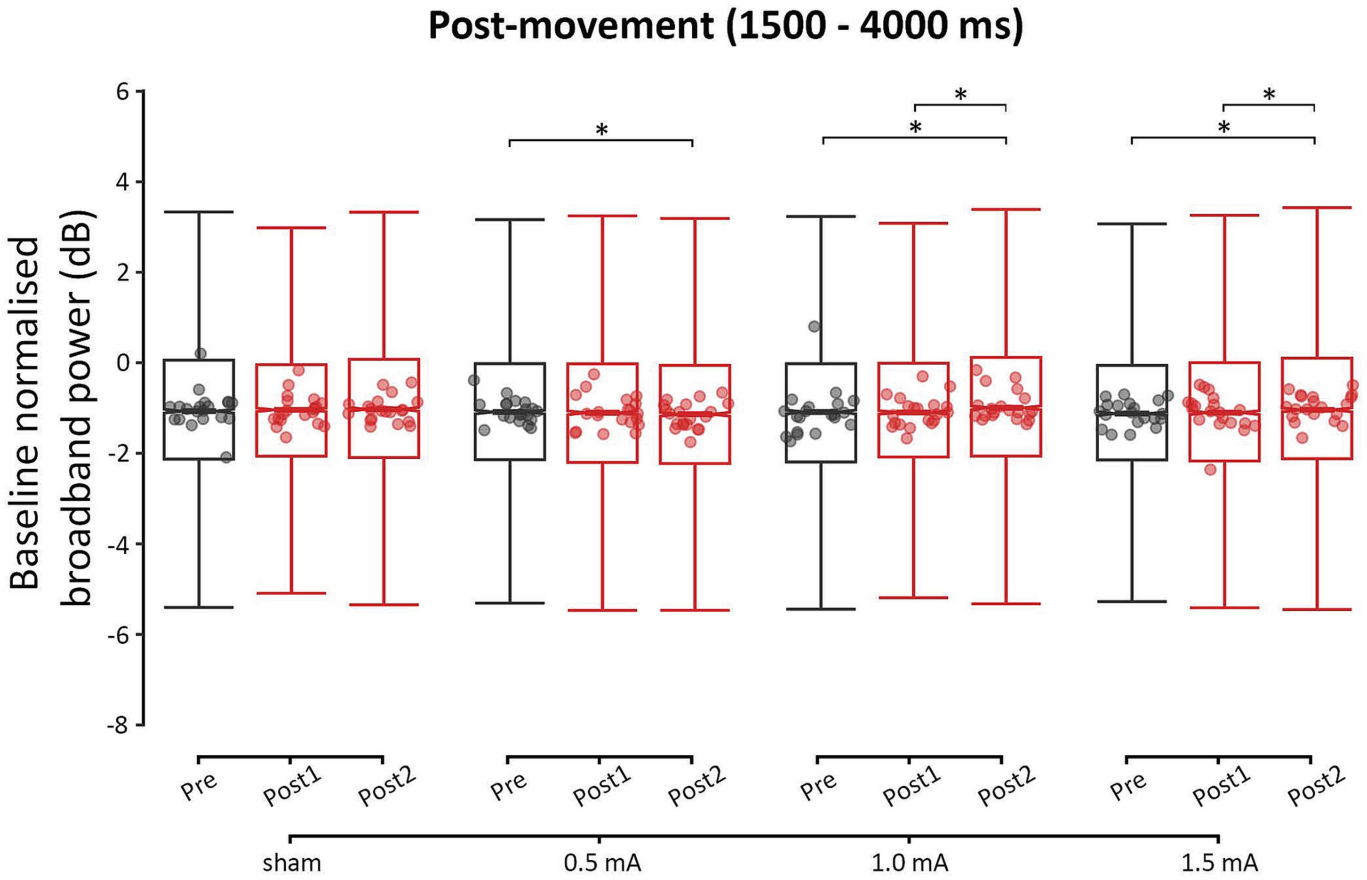
Event-related changes bilateral broadband power (4–90 Hz) of the post-movement period (1,500–4,000 ms), for each stimulation intensity. Power values have been baseline-normalized to the period of −2,000 to −1,000 ms. * = significant change between time-points at α = 0.05. Data points reflect participant averages. The height of the notches reflects the median ± 1.57 × IQR/sqrt(n) where IQR is the interquartile range defined by the 25th and 75th percentiles and n is the number of data points.

Figure 6 shows the post-movement power values for each time-point, and stimulation intensity. Unlike the other two movement periods, there were no significant differences between the stimulation intensities at post-movement baseline. Tables of these results can be found in the Supplementary Results. From pre to post_1_, none of the four stimulation intensities showed a significant change in post-movement power (|*z*s| ≤ 1.296, *p*s ≥ 0.397, |*d*s| ≤ 0.001). From pre to post_2_ and from post_1_ to post_2_, there were significant increases in post-movement power following 1.0 mA and 1.5 mA stimulation (|*z*s| ≥ 3.803, *p*s < 0.001, |*d*s| ≥ 0.004), but there was no difference between these intensities in the magnitude of power increase (|*z*s| ≤ 0.468, *p*s = 1.000, |*d*s| ≤ 0.068). These increases were likely induced by tACS, as changes were not observed following sham stimulation (*z* = −0.591, *p* = 0.825, *d* = −0.001). In contrast, 0.5 mA stimulation showed a significant decrease in post-movement power from pre to post_2_ (*z* = 2.460, *p* = 0.037, *d* = 0.002), but no change from post_1_ to post_2_ was observed (*z* = 2.034, *p* = 0.104, *d* = 0.002).

### 3.4 Changes in peak beta frequencies

We also examined each movement period to determine whether any of the stimulation intensities caused a shift in participants’ peak beta frequencies. Specifically, we investigated whether tACS shifted the endogenous-exogenous frequency difference (i.e., the difference between participants’ endogenous peak beta frequencies and the exogenous tACS frequency of 20 Hz). The GLMM analyses revealed a significant main effect for REGION in the pre- and post-movement periods (*χ*^2^s ≥ 4.824, *p*s ≤ 0.028), and a significant main effect for TIME in the movement period (*χ*^2^ = 23.976, *p* < 0.001). All other main effects were non-significant (*χ*^2^s ≤ 6.756, *p*s ≥ 0.080). The analyses also revealed a significant two-way INTENSITY × TIME interaction for the movement period (*χ*^2^ = 15.372, *p* = 0.018). All other interactions were non-significant (*χ*^2^s ≤ 10.806, *p*s ≥ 0.095). These results indicate that tACS induced stimulation intensity-specific changes in the endogenous-exogenous frequency differences of the movement period, but not of the pre- and post-movement periods. The highest level of interaction—the two-way INTENSITY × TIME interaction of the movement period—was further investigated with *post-hoc* comparisons.

**Figure 7:**
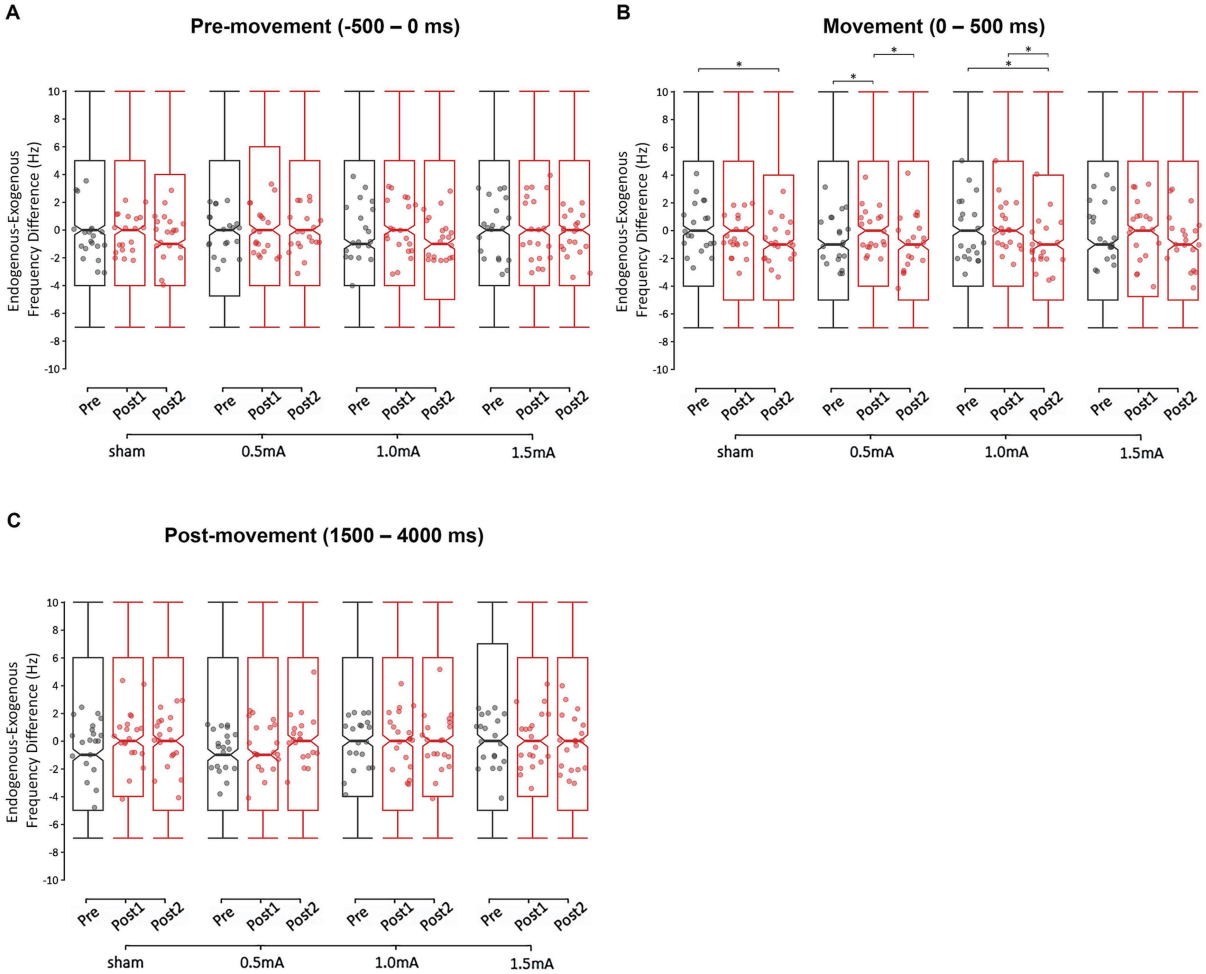
Changes in the endogenous-exogenous frequency difference at each stimulation intensity, for the **(A)** pre-movement (−500 to 0 ms), **(B)** movement (0–500 ms), and **(C)** post-movement (1,500–4,000 ms) periods. The endogenous-exogenous frequency difference was calculated by subtracting the stimulation frequency (20 Hz) from each participant’s peak beta frequency. * = significant change between time-points at α = 0.05. Data points reflect participant averages. The height of the notches reflect the median ± 1.57 × IQR/sqrt(n) where IQR is the interquartile range defined by the 25th and 75th percentiles and n is the number of data points.

Figure 7 shows the endogenous-exogenous frequency differences for each time-point, stimulation condition, and movement period. There was a significant baseline difference only between 0.5 mA and sham stimulation (*z* = −3.137, *p* = 0.010, *d* = −0.049), so comparisons between these two intensities should be interpreted with caution. From pre to post_1_, 0.5 mA stimulation showed a significant increase in the frequency difference of the movement period (*z* = −2.546, *p* = 0.033, *d* = −0.040) from 0.117 to 0.447 Hz. From pre to post_2_, 1.0 mA stimulation showed a significant decrease in the frequency difference (*z* = 3.716, *p* < 0.001, *d* = 0.059), from 0.726 to −0.031 Hz. This decrease was unlikely due to tACS, as a decrease was also observed following sham stimulation (*z* = 2.902, *p* = 0.011, *d* = 0.045), and there was no difference in the magnitude of decrease following 1.0 mA and sham stimulation (*z* = 0.610, *p* = 1.0000, *d* = 0.277). From post_1_ to post_2_, both 0.5 mA and 1.0 mA stimulation showed significant decreases in frequency differences (i.e., closer to zero; |*z*s| ≥ 2.774, *p*s ≤ 0.017, |*d*s| ≥ 0.045), but there was no difference between conditions (*z* = 0.868, *p* = 1.000, *d* = 0.398). There were no other significant changes in the endogenous-exogenous frequency difference of the movement period (|*z*s| ≤ 1.834, *p*s ≥ 0.200, |*d*s| ≤ 0.029).

### 3.5 Changes in event-related power were associated with pre-tACS endogenous-exogenous frequency differences

**Figure 8:**
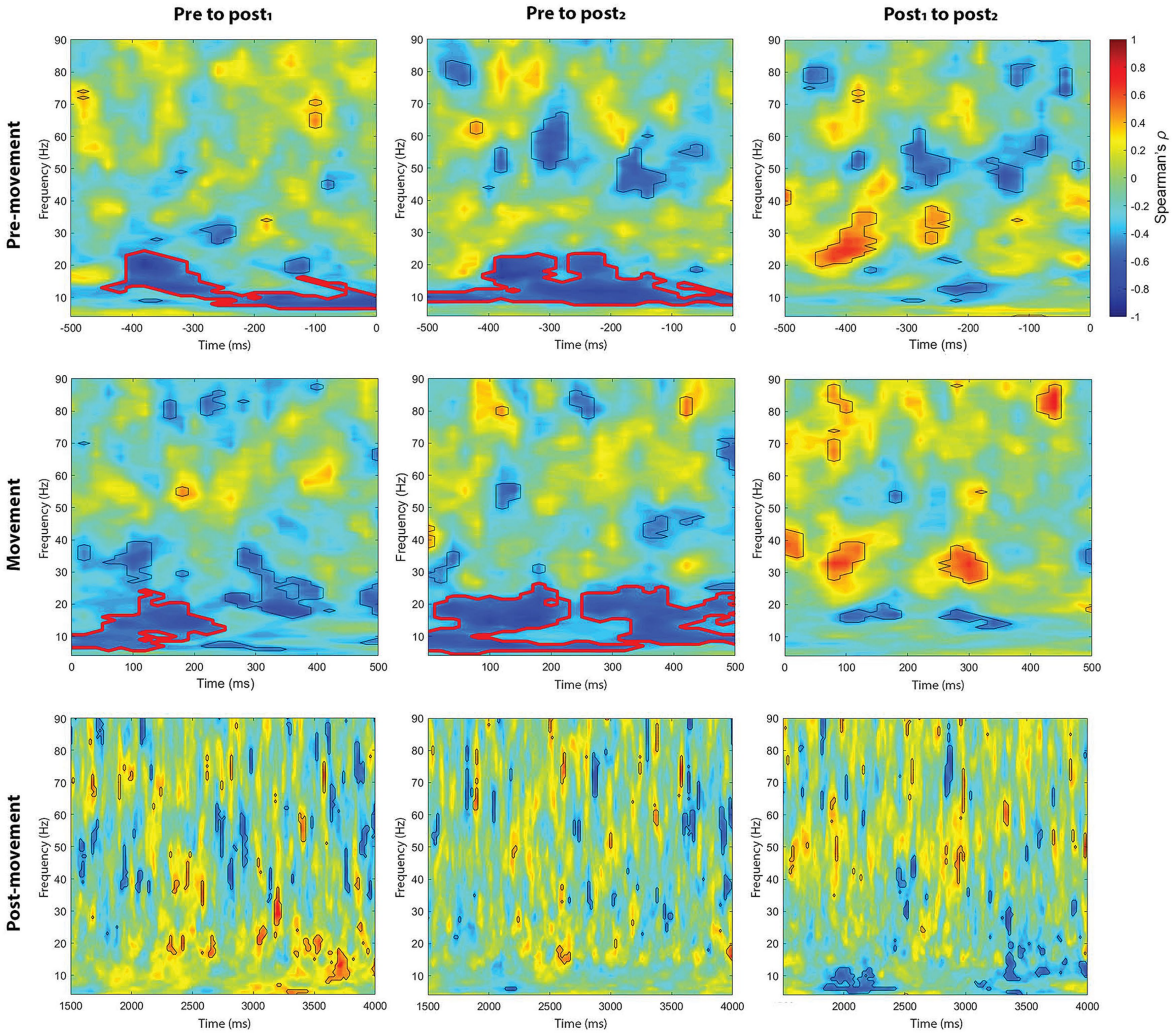
Cluster-based correlations (Spearman’s *ρ*) between endogenous-exogenous frequency differences and changes in event-related power, following 1.0 mA stimulation. Areas bordered with thick red lines indicate clusters with significant correlations.

**Figure 9:**
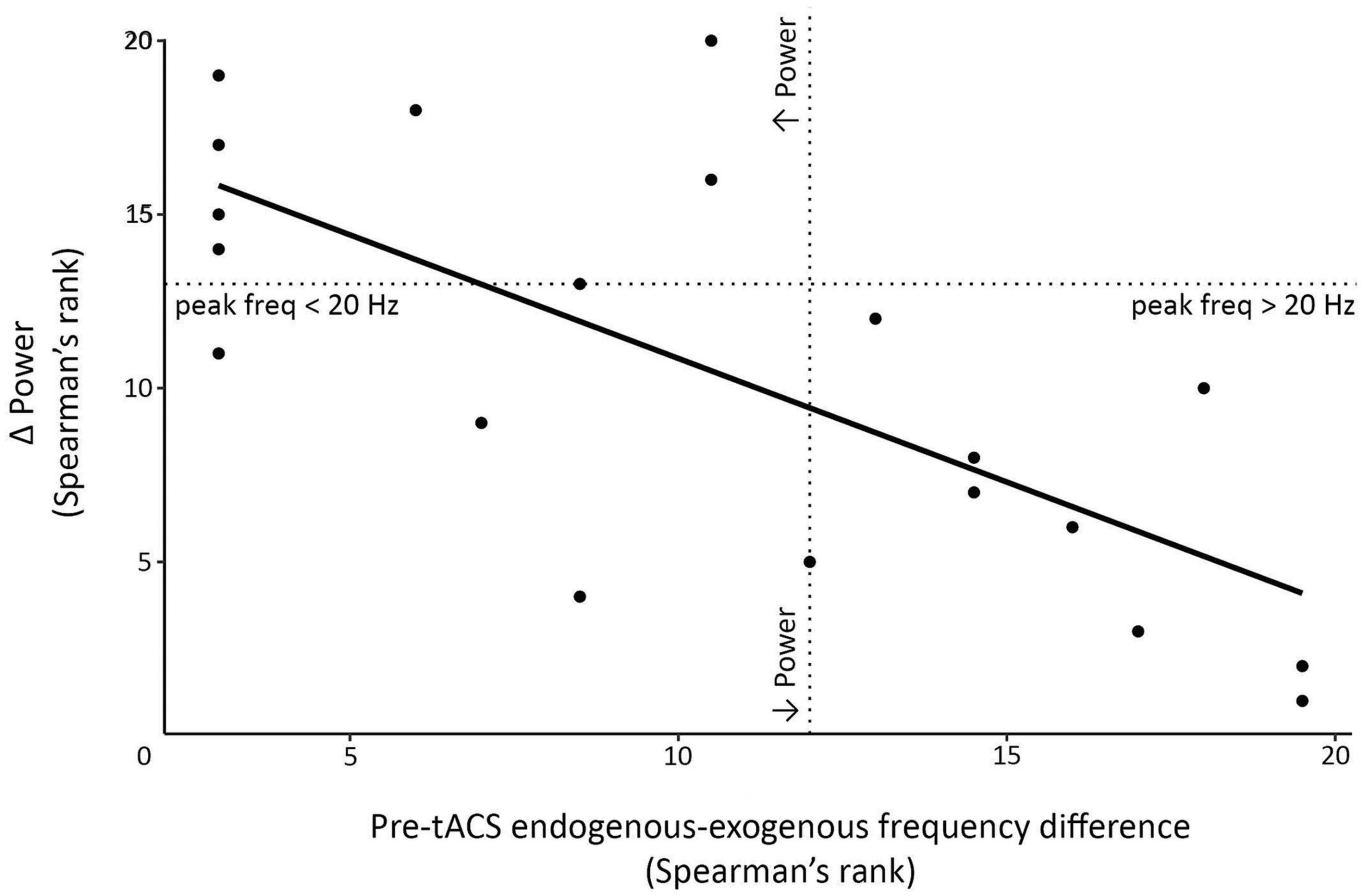
Scatterplot demonstrating significant negative correlation clusters observed following 1.0 mA stimulation. Correlations assessed the relationship between the pre-tACS endogenous-exogenous frequency difference and the post-tACS change in event-related power (*Δ* power). In this example, data were extracted from the pre-movement period, at a time-frequency point within the significant cluster (specifically, from pre to post_1_ at 300 ms and 15 Hz). Dotted lines indicate points where the endogenous-exogenous frequency difference and/or Δ power equals 0.

We also explored whether the endogenous-exogenous frequency differences were associated with the change in event-related power following tACS. We performed separate cluster-based correlations on each movement period, time-point comparison, stimulation intensity, and region. From pre to post_1_ and from pre to post_2_, there were significant negative correlations in the pre-movement period following 1.0 mA stimulation at the C3 electrode cluster. From pre to post_1_, the significant negative cluster was between 7 Hz to 24 Hz and between −440 to 0 ms (see Figure 8). From pre to post_2_, the significant negative cluster was between 8 Hz to 23 Hz and between −500 to 0 ms. To better understand the nature of these correlations, we generated a scatterplot of endogenous-exogenous frequency differences and *Δ* event-related power values at several time-frequency points within the clusters (see Figure 9 for an example). Relative to an endogenous-exogenous frequency difference of 0 Hz, the results were bidirectional, suggesting that individuals with peak frequencies further below 20 Hz showed greater increases in power following 1.0 mA tACS, and individuals with peak frequencies further above 20 Hz showed greater decreases in power following 1.0 mA tACS.

Similar results were observed in the movement period. From pre to post_1_ and from pre to post_2_, there were significant negative correlations in the movement period following 1.0 mA stimulation at the C3 electrode cluster. From pre to post_1_, the significant negative cluster was between 6 Hz to 24 Hz and between 0 to 240 ms (see Figure 8). From pre to post_2_, the significant negative clusters were between 5 Hz to 26 Hz and between 0 to 500 ms. As with the pre-movement period, the negative movement period correlations were bidirectional.

There were no significant clusters for any other stimulation intensity in the pre-movement or movement periods, or any significant clusters for the post-movement period at C3 post-tACS. Additionally, no significant clusters were observed for any movement period or stimulation condition at the C4 electrode cluster post-tACS. Together, these results suggest that participants’ endogenous beta frequency was associated with the aftereffects of 1.0 mA stimulation in the pre-movement and movement periods, over the stimulated region.

## 4 Discussion

Here, we comprehensively investigated the effect of beta tACS, positioned over left M1 at different stimulation intensities, on resting-state and event-related sensorimotor power. For the resting-state, all stimulation, including sham, lead to increases in theta, alpha, and beta power at the left M1 ROI. Further, both 1.0 mA and 1.5 mA stimulation produced distinct hemispheric changes in resting-state beta power. For event-related power, we did not observe a frequency specific change in beta activity. Instead, intensity-dependent changes were observed in broadband power (4–90 Hz). For the pre-movement period, 1.0 mA stimulation increased power. For the post-movement period, 0.5 mA stimulation decreased power while 1.0 mA and 1.5 mA stimulation induced comparable increases in power. None of the stimulation intensities induced changes in broadband power during movement, though 1.0 mA stimulation shifted the peak beta frequency of this movement phase toward the exogenous tACS frequency. Notably, changes in pre-movement and movement power following 1.0 mA stimulation were negatively associated with participants’ pre-tACS peak beta frequency.

### 4.1 Effect of tACS on resting-state power

#### 4.1.1 All stimulation intensities increased theta, alpha, and beta power at the left M1 ROI

All stimulation intensities, including sham, showed increases across all frequency bands, except gamma, from pre to post_1_ (~5 min post-tACS) and from pre to post_2_ (~25 min post-tACS). These power increases were unlikely due to tACS, as the extent of changes following real stimulation was not greater than the changes following sham stimulation. There are four possible explanations for this broad (4–30 Hz) power increase. First, the observed changes in power may simply reflect spontaneous fluctuations in EEG activity. Second, these changes in power may be attributed to improvements in the signal-to-noise ratio from the pre- to post-tACS measurements. This phenomenon is frequently encountered in EEG research (Kappenman and Luck, 2010). As the signal-to-noise ratio increases, the true neural signal becomes more prominent relative to background noise, potentially leading to apparent increases in power across frequency bands. Third, the increase in resting-state power might have been due to repeated activation of the sensorimotor cortex during the movement recordings, which involved ~60 self-paced index finger flexions over 10 min. However, the absence of change in resting-state power in sham from post_1_ to post_2_ indicates that this was unlikely. The post_1_ resting-state measurement followed a long (>20 min) period of inactivity, while the post_2_ resting-state measurement immediately followed a movement block. If the movement caused the broad increase in resting-state power, it is likely that we should have seen an increase between post_1_ and post_2_ within the sham condition. Indeed, though changes in resting-state power have been observed following substantial motor training (e.g., Moisello et al., 2015), studies that have implemented simple movement protocols, similar to the current study (without tACS), have not observed changes in resting-state power (e.g., Dornowski et al., 2018; Sasaki et al., 2018). Fourth, the increase in resting-state power might have been due to mental fatigue. Each experimental session lasted 2–2.5 h, and some participants anecdotally reported feeling fatigued during both post-tACS EEG recordings. A recent meta-analysis showed that mental fatigue was positively associated with a broad (1–30 Hz) increase in EEG power, similar to what was observed in the current study, which reflect homeostatic processes (Tran et al., 2020). We considered additional analyses to further investigate whether these broad power increases might be related to fatigue accumulation across sessions (i.e., if beta power increases were greater in later sessions), but did not pursue this due to statistical power limitations. Our counterbalanced design resulted in only 5–6 participants receiving sham in any given sequence position, providing insufficient statistical power for reliable analysis of order-specific effects. We also lacked standardized pre/post fatigue measurements that would allow us to quantify fatigue development throughout each session or correlate it with the observed power increases. Future studies comprising larger sample sizes should consider taking measures of fatigue (e.g., heart rate variability or self-report questionnaires) to account for this variable.

#### 4.1.2 The frequently used tACS intensity of 1.0 mA did not induce a greater increase in beta power of the left M1 ROI compared to sham

When applying tACS at 1.0 mA, numerous studies have observed significant post-stimulation increases in resting-state M/EEG power of the stimulated frequency band (e.g., Battaglini et al., 2020; D’Atri et al., 2019; Zaehle et al., 2010). Accordingly, we expected that 1.0 mA tACS (but not sham) would increase resting-state beta power. However, we found that the increase in beta power following 1.0 mA stimulation was comparable to the increase in power following sham stimulation. There are a few possible explanations for the non-significant difference between 1.0 mA and sham stimulation.

Although we did not record EEG during tACS, it is possible that the combined frequency and current intensity of tACS used in this study were not adequate to effectively modulate beta oscillations during the stimulation period. Results from rodent studies suggest that stimulation at frequencies >10 Hz might require higher current intensities to induce a response (e.g., Anastassiou et al., 2011; Deans et al., 2007). This might explain the discrepancy between the null finding in the current study and significant findings in previous human M/EEG studies, where 1.0 mA tACS was applied at ≤10 Hz. However, this notion is contradicted by evidence of 1.0 mA beta tACS inducing significant changes (e.g., greater motor learning: Pollok et al., 2015). With conflicting findings, it remains unclear as to whether the tACS protocol used in the current study was able to effectively modulate the neural oscillations of the left M1 ROI during the stimulation period. If real tACS modulated neural activity as it was delivered, the effect might not have outlasted the stimulation period. This study was limited to recording EEG before and after stimulation because, during stimulation, the tACS electrodes obstruct the EEG electrodes that record activity from the target site and the tACS stimulation artifact contaminates activity recorded from nearby electrodes (Asamoah et al., 2019; Kasten and Herrmann, 2019; Neuling et al., 2017; Noury et al., 2016; Noury and Siegel, 2018). Future studies might overcome this limitation with devices that support concurrent stimulation and recording, as well as developments in artifact removal techniques (Fehér and Morishima, 2016).

Another possible explanation is that real tACS induced a lasting effect on left M1 neural activity, but the measures used in the current study were unable to capture the effect. A recent *in vivo* study of non-human primates demonstrated that tACS could entrain the firing of individual neurons (Johnson et al., 2020). However, across all stimulation intensities (0.5 mA, 1.0 mA, and 1.5 mA), a maximum of 9 neurons out of 34 were simultaneously entrained, demonstrating that only a relatively small proportion of neurons responded to tACS. Measuring changes in left M1 neural activity non-invasively with EEG power requires a large population of neurons to be modulated in order to be recognized. Therefore, in the current study, it is possible that real tACS modulated neural activity beyond the duration of stimulation, but the extent of neuromodulation was not in a sufficiently large population of neurons to be captured by EEG power spectral analysis. Our results did, however, suggest that 1.0 mA beta tACS might have influenced areas that include the M1-M1 network. While sham stimulation resulted in similar increases in beta power at both the left and right M1 ROIs, 1.0 mA stimulation produced distinct regional differences. Specifically, beta power increased in the left M1 ROI but decreased in the right M1 ROI, suggesting stimulation-dependent changes in the M1-M1 network. This interpretation was supported by follow-up EEG connectivity analyses, reported in Wansbrough et al. (2024a), which demonstrated that 1.0 mA stimulation significantly reduced M1-M1 connectivity compared to sham. These findings suggest that 1.0 mA beta tACS might reduce coupling between the stimulated left M1 ROI and the unstimulated right M1 ROI. Given the potential functional implications of altered interhemispheric connectivity, future research should investigate the functional consequences of unifocal tACS on both unilateral and bilateral movement.

Finally, the null-finding here might be explained by both intra- and inter-individual variability. Between-session intra-individual variability contributed to baseline differences in resting-state and event-related power. The GLMMs accounted for this variability by incorporating participant-specific random intercepts, allowing for appropriate modeling of individual differences. Given the centered random intercepts, intra-individual variability is not believed to have significantly biased the interpretation of tACS-induced changes. Additionally, the results revealed substantial inter-individual differences in tACS response. For example, at post_1_, ~50% of participants showed a decrease in resting-state beta power at left M1 ROI following 1.5 mA stimulation, while ~25% showed an increase and ~25% showed no change (defined as <10% change). This inter-individual response variability is a common issue in NIBS literature (for a review, see Guerra et al., 2020). Various factors may contribute to inter-individual response variability, including differences in anatomical, neurochemical, and demographic characteristics (for a review of these factors, see Vergallito et al., 2022). Consequently, despite applying identical intensities across participants, variations in skull thickness, cortical folding patterns, and tissue conductivity properties likely resulted in inter-individual differences in electric field distributions reaching neural targets (Hunold et al., 2023). Another potential source of inter-individual variability relates to dynamic systems theory (Pikovsky et al., 2001), which suggests that the alignment between endogenous oscillations and the exogenous stimulation frequency influences entrainment outcomes. We made an effort to reduce the impact of inter-individual variability by implementing a within-subjects design, recruiting a sample of healthy right-handed young adults, and by taking individual differences into consideration in the statistical models as a random intercept. However, sources of variability would have still impacted the individual response to tACS, which might have contributed to the null finding. Future studies may overcome these issues through individualized stimulation approaches that account for both anatomical differences (through electric field modeling) and oscillatory characteristics (through frequency-calibrated stimulation) (Wansbrough et al., 2024b).

Importantly, our results align with a growing body of literature demonstrating inconsistent effects of beta tACS on sensorimotor oscillations. While several studies have reported significant increases in beta power following 20 Hz stimulation (Battaglini et al., 2020; Lyzhko et al., 2023; Nakazono et al., 2020; Suzuki et al., 2022), others have failed to observe significant modulation (Akaiwa et al., 2024; Harada et al., 2020; Lafleur et al., 2021; Rumpf et al., 2019; Sugata et al., 2018). These discrepancies may be related to methodological variations including electrode montages, stimulation durations, and measurement approaches. These reproducibility challenges underscore the need for more systematic parameter investigations and standardized methodological approaches in beta tACS research. Multi-site studies with standardized protocols could provide important insights into the reproducibility of beta tACS effects, both physiologically (on neural oscillations) and functionally (on motor performance). Additionally, reporting individual-level data alongside group-level analyses may help identify factors contributing to response variability and inform more reliable stimulation protocols. Limited evidence of intensity-dependent changes in resting-state power of the target frequency (beta) band.

Previous *in vivo* research of both rodents and non-human primates has shown that the entrainment of neural firing depends on the stimulation intensity. When applying tACS at a set frequency, higher stimulation intensities entrained more neurons to fire at the tACS frequency compared to lower intensities (Asan et al., 2020; Huang et al., 2021; Johnson et al., 2020; Krause et al., 2022). Several human studies have also demonstrated intensity-dependent changes in EEG power of the stimulated frequency band (De Koninck et al., 2021; Wang Y. et al., 2022), as well as changes in corticospinal excitability (Moliadze et al., 2012; Shorafa et al., 2021). The hypothesis of linear intensity-dependent changes in resting-state beta power at the left M1 ROI was not supported by the changes in beta power, which did not significantly differ between intensities. This non-significant finding might be explained by the reasons discussed in the previous section. Interestingly, the previously noted region-specificity of the tACS response appeared to be intensity-specific: increases in beta power were significantly different between the stimulation site (left M1 ROI) and the contralateral site (right M1 ROI), following only 1.0 mA and 1.5 mA stimulation.

### 4.2 Effect of tACS on event-related power

#### 4.2.1 The frequently used tACS intensity of 1.0 mA did not induce greater changes in event-related beta power compared to sham

Only three studies have previously examined the effect of beta tACS (when applied at rest over the left M1) on event-related M/EEG power (Akaiwa et al., 2024; Harada et al., 2020; Sugata et al., 2018). Following 1.0 mA beta tACS, no changes event-related beta power were observed during movement (Harada et al., 2020; Sugata et al., 2018), or post-movement (Akaiwa et al., 2024). In the current study, 1.0 mA stimulation did not selectively modulate the beta ERD/ERS of any movement period. Instead, there was a broadband increase (4–90 Hz) in pre-movement and post-movement power, but not movement power. The pre- and post-movement increases in power were likely due to tACS, as sham stimulation did not show the same effect. It is unclear why responses within each movement period differed. One possible explanation might be that the brain state underlying each movement period affected the response to tACS. Indeed, these movement periods are underpinned by slightly different neural processes (e.g., differences in excitation/inhibition; Kilavik et al., 2013), and research suggests that differences in brain states can affect the tACS response (e.g., Shorafa et al., 2021; Wang Y. et al., 2022). Interestingly, the increases in pre- and post-movement power were delayed, being only observed at post2 (~25 min post-tACS). The delayed responses may reflect late plasticity-like mechanisms, which have been observed in corticospinal excitability following 20 and 250 Hz tACS (Moliadze et al., 2010). Further, current results did not show a significant effect of region, indicating that 1.0 mA tACS might have had a similar effect on the stimulated left M1 ROI and the non-stimulated right M1 ROI. The pre-movement ERD and post-movement ERS are observed bilaterally (Kilavik et al., 2013), thus, the bilateral response observed following tACS might reflect a functional spread of neuromodulation. Further research is needed to clarify the exact mechanisms underpinning the observed effect of tACS on movement-related neural activity, as well as the functional effects of these changes. Beta tACS did not modulate event-related peak beta frequencies.

#### 4.2.2 Evidence of intensity-dependent changes in event-related power

While we did not see any tACS-induced changes specific to the beta band, we observed intensity-dependent changes in broadband (4–90 Hz) power of the pre- and post-movement periods, but not the movement period. In the pre-movement period, 1.0 mA stimulation induced a delayed increase in power (from pre to post_2_), while sham, 0.5 mA and 1.5 mA stimulation showed no change. For post-movement power, we observed significant increases in power following both 1.0 mA and 1.5 mA stimulation (from pre to post_2_ and from post_1_ to post_2_), though the extent of changes did not significantly differ between 1.0 mA and 1.5 mA stimulation, indicating that there might have been a plateau. Together, these results indicate that tACS induced a non-linear dose–response effect on pre- and post-movement power. The non-linear response is somewhat in line with the findings of De Koninck et al. (2021), who found that alpha tACS at 1.0 mA induced a greater increase in alpha power compared to stimulation at 4.0–6.0 mA. The authors suggested that the non-linear intensity-dose response might be explained by compensatory or homeostatic mechanisms – a finding that is also observed in transcranial direct current stimulation (Batsikadze et al., 2013). It is unclear why we observed broadband effects rather than beta-specific ones; this may be related to cross-frequency coupling mechanisms, which we discuss in detail in the Supplementary Material along with the additional results within other frequency bands.

Interestingly, although there was no significant change in post-movement power following sham stimulation, there was a significant decrease in power following 0.5 mA stimulation (from pre to post_2_). This finding might have been a result of spontaneous fluctuations or might suggest that 0.5 mA beta tACS induced a suppression of power. Suppressed EEG power following 0.5 mA tACS aligns with the results from a recent study in non-human primates. Krause et al. (2022) found that applying tACS at a lower intensity decreased entrainment of neural firing. The authors reasoned that the weak current was unable to override the rhythm of neural firing, resulting in a competition for control between the natural endogenous rhythm and the exogenous current. Similar mechanisms might explain the suppression observed in the current study, though it is not clear why suppression occurred across a broad range of frequencies. Indeed, Krause et al. (2022) only observed effects within a narrow window around the stimulation frequencies. Comparisons between these two studies should be taken with caution as there are significant methodological differences between them. For example, Krause et al. (2022) analyzed local field potentials recorded during tACS. Without more comprehensive and extensive analyses, the exact mechanisms underpinning the suppression of post-movement in this study remain uncertain.

We also observed instances where power values decreased following sham stimulation, but not real stimulation. For example, in the pre-movement period, there was a decrease in power following sham stimulation from post_1_ to post_2_, but no changes following 0.5 mA, 1.0 mA, or 1.5 mA stimulation. A speculative interpretation of this might be that the real stimulation promoted stability of sensorimotor oscillations, though the mechanisms underlying this are unknown. Overall, while tACS did not appear to selectively modulate pre-movement, movement, and post-movement beta oscillations, the results suggest that tACS can modulate broad sensorimotor activity, and that the stimulation intensity is an important consideration for the modulation of this activity. While the current study did not directly assess behavioral outcomes, the observed intensity-dependent changes in broadband power may have functional significance. The increases in pre-movement power following 1.0 mA stimulation suggest that this protocol could potentially influence motor readiness and response selection (Kilavik et al., 2013). Similarly, the bidirectional modulation of post-movement power (increases after 1.0 mA and 1.5 mA; decreases after 0.5 mA) may affect motor inhibition processes, which are critical for movement termination (Heinrichs-Graham and Wilson, 2016). Notably, the fact that different movement phases showed distinct responses to stimulation suggests that adaptive stimulation protocols, which take specific movement phases into account, might be necessary to achieve targeted functional effects. Future studies incorporating behavioral measures are needed to directly test these hypotheses and determine the optimal stimulation parameters for specific functional outcomes. Additionally, future research should test a broader range of intensities, particularly above 1.5 mA, to provide a more comprehensive understanding of the dose–response relationship. Several studies have already explored higher tACS intensities: De Koninck et al. (2021) found low intensity (1.0 mA) stimulation induced greater and longer-lasting increases in alpha power than higher intensities (4.0–6.0 mA); Wang Y. et al. (2022) demonstrated that 2.0 mA alpha tACS increased resting-state alpha power more effectively than 1.0 mA, but only when participants had their eyes open; and clinical work by Wang H. et al. (2022) successfully applied 15 mA currents in depression treatment. Similarly, investigating different stimulation durations (e.g., >20 min) could help determine whether longer stimulation periods produce stronger or more persistent effects. Indeed, extended protocols have shown promising outcomes, such as Del Felice et al.’s (2019) 30 min tACS sessions for Parkinson’s disease, which modulated sensorimotor oscillations and improved motor performance. However, there remains a need for systematic investigation directly comparing different stimulation durations. Such extended parameter exploration would be especially valuable given the non-linear intensity-dependent effects observed in our study and others, and would contribute to developing more effective tACS protocols for both research and potential clinical applications.

### 4.3 Only 1.0 mA beta tACS shifted participants’ event-related endogenous-exogenous frequency differences

To further explore the effect of beta tACS, we examined whether participants’ peak beta frequency of each movement period shifted following stimulation by analyzing the change in the endogenous-exogenous frequency difference. For the pre-movement and post-movement periods, the endogenous-exogenous frequency difference did not significantly change following tACS. However, for the movement period, there were several significant changes in the endogenous-exogenous frequency difference following tACS. From pre to post_1_, 0.5 mA stimulation significantly shifted participants’ endogenous peak beta frequency further away from the exogenous frequency (from 0.117 to 0.447 Hz)—this may reflect the lower intensity stimulation disrupting beta activity, as discussed in section 3.6.2.2. The difference then shifted back toward baseline by post_2_. In contrast, the endogenous-exogenous frequency difference remained stable from pre to post_1_ following 1.0 mA tACS, but significantly shifted closer to zero at post_2_ (from 0.726 to −0.031 Hz). This finding indicates that 1.0 mA stimulation induced a delayed shift in peak beta toward the exogenous tACS frequency. This finding emphasizes the value of considering the shift in peak frequency as an effect of tACS. It will be important for future research to examine the functional effect of this shift, and whether similar shifts occur within other frequency bands.

### 4.4 Changes in event-related power following 1.0 mA stimulation were associated with pre-tACS endogenous-exogenous frequency differences

In an effort to improve the efficacy of tACS, some studies have individualized the stimulation frequency to participants’ peak frequency. For example, Ayanampudi et al. (2023) found that individualized theta/alpha tACS induced superior improvements in sleep quality compared to both sham and fixed frequency tACS. If individualized frequencies induce superior neuromodulation, it is plausible that the participants’ pre-tACS endogenous-exogenous frequency difference might have been associated with the change in event-related power post-tACS. For both the pre-movement and movement periods, we observed significant relationships between participants’ pre-tACS endogenous-exogenous frequency difference and the changes in power following 1.0 mA tACS. Significant associations were evident at both post_1_ and post_2_, though they were specific to activity at the stimulated left M1 ROI and changes in the frequency range of 5–26 Hz. It is unclear why the relationship was only significant for these two movement periods and only following 1.0 mA stimulation. Interestingly, the relationships were bidirectional, suggesting that individuals with peak beta frequencies further below 20 Hz showed greater increases in power post −1.0 mA tACS, and individuals with peak frequencies further above 20 Hz showed greater decreases in power post −1.0 mA tACS. These findings align with Kudo et al. (2022), who observed a negative correlation between baseline endogenous-exogenous frequency differences and the changes in corticomuscular coherence induced by 20 Hz oscillatory tDCS. Together, these findings indicate that there may be a complex interaction between the stimulation intensity and frequency to modulate electrophysiological function. Structural factors such as variations in sensorimotor cortical thickness and white matter microstructure could affect conduction velocities and oscillation frequencies (Hunt et al., 2016). Physiologically, variability in excitation/inhibition balance (Gaetz et al., 2011; Wessel et al., 2016), intrinsic neuronal properties including membrane time constants and ion channel expression (Buzsáki et al., 2012), and thalamocortical connectivity patterns (Sherman et al., 2016), may all contribute to individual variations in peak beta frequencies. Future research should systematically investigate these anatomical, physiological, and experiential factors that determine individual peak beta frequencies to better understand and predict the neurophysiological and motor behavioral responses to individualized beta tACS, compared to fixed frequency beta tACS.

### 4.5 Limitations

The current study was limited to a single frequency (20 Hz) in assessing tACS effects on sensorimotor power, leaving the specificity of intensity-dependent changes to beta stimulation unresolved. Given the documented frequency-dependent effects of tACS over left M1 on corticospinal excitability and motor function (for a review, see Rostami et al., 2024), future research should explore whether such frequency-dependence extends to resting-state and event-related sensorimotor power. This could involve comparing connectivity changes across various stimulation frequencies, including those prominently associated with motor function, such as alpha and gamma (Pfurtscheller and Lopes Da Silva, 1999).

Furthermore, our choice of 20 Hz stimulation sits at the boundary between low beta (13–20 Hz), which is predominantly associated with movement preparation and post-movement inhibition, and high beta (20–30 Hz), which is more closely related to motor control, movement execution, and sensorimotor processing (Nougaret et al., 2024). This boundary position might partly explain the lack of frequency-specific effects on event-related beta activity observed in our study. While we selected 20 Hz to enable direct comparison with previous resting-state beta tACS studies (as it is the most commonly used frequency in this literature), future research should consider systematically investigating the differential effects of low versus high beta tACS on event-related sensorimotor activity to provide greater insight into the frequency-specific mechanisms underlying sensorimotor beta oscillations.

Another important consideration relates to our study’s exclusive use of a HD montage, which raises questions about the generalizability of the observed effects to conventional bipolar montages. Considering the differences in electric field spread between these montages (Datta et al., 2008; Dmochowski et al., 2011) and the established link between field spread and neuromodulation outcomes (Hunold et al., 2023), further investigation is warranted. For instance, Kasten et al. (2019) demonstrated that individual variability in tACS-induced alpha power modulation correlates with modeled electric field spread. These findings suggest that future studies should examine whether the changes observed here persist when tACS is applied using a conventional bipolar montage. Further, despite the improved focality of a HD montage (relative to a bipolar montage), HD-tACS still suffers from shortcomings in spatial specificity. While our montage was designed to maximize current density at the left M1, the stimulation inevitably affects surrounding regions (Alekseichuk et al., 2019; Kasten et al., 2019), limiting our ability to attribute effects solely to M1 stimulation.

Cognitive state during tACS is an important consideration as neural oscillations, particularly in the beta band, are highly sensitive to attentional load and cognitive engagement (Engel and Fries, 2010; Spitzer and Haegens, 2017). In the current study, participants were instructed to keep their eyes open while the researcher engaged them in light conversation during the 20 min stimulation. This approach helped maintain alertness and prevent drowsiness, but introduced variability in the exact content and level of cognitive engagement across participants and sessions. Alternative approaches include having participants perform vigilance tasks (e.g., Antal et al., 2008; Kasten et al., 2016), watch neutral videos (e.g., Alexander et al., 2019), or fixate on a cross in silence (Neuling et al., 2013). While these methods offer greater standardization, they may still lead to variable attentional states or, in the case of passive fixation, increased drowsiness over extended periods. The interaction between brain state and tACS efficacy is well-documented, with several studies demonstrating that the same stimulation parameters can produce different outcomes depending on underlying brain activity (e.g., Alagapan et al., 2016; Neuling et al., 2013; Shorafa et al., 2021; Wang Y. et al., 2022). Currently, no single approach has emerged as best practice. Future research should systematically compare different cognitive engagement protocols during stimulation to determine how various approaches affect the reliability and magnitude of tACS effects and to establish optimized standardization guidelines.

An additional limitation of our study is the focus on only two ROIs (left and right M1s). Although our bilateral analysis provides valuable insights into spatial specificity of tACS effects, future research should examine how M1 stimulation influences oscillatory activity in other functionally connected regions, such as the supplementary motor area (SMA) and the parietal cortex. Such investigations would require larger sample sizes to maintain adequate statistical power while comparing multiple regions.

A related limitation concerns statistical power given our sample size. A *post-hoc* power analysis based on model t-statistics revealed that our study (*N* = 21) had adequate power (≥0.80) to detect all main effects (INTENSITY: 0.81, TIME: 1.00, FREQUENCY: 1.00, REGION: 1.00) and two key two-way interactions (TIME × REGION: 0.91, INTENSITY × REGION: 0.84), but had limited power to detect more complex interaction patterns. Three-way and four-way interactions showed considerably lower power levels (0.20–0.63), a common issue in neurophysiological studies with complex factorial designs (Button et al., 2013). This suggests that our findings regarding main effects and key two-way interactions are statistically robust, while more complex interaction patterns should be interpreted with appropriate caution. The significant higher-order interactions we did detect likely represent particularly strong effects considering the conservative nature of our analysis. Future studies examining these complex interaction patterns would benefit from larger sample sizes (approximately 60–80 participants based on our power calculations) to achieve adequate power across all levels of interaction.

Finally, our study was limited by the sensations induced by tACS. Analysis of our sensation questionnaire data revealed that participants experienced significantly stronger sensations during 1.0 mA and 1.5 mA stimulation compared to sham. While some research in animal models suggests that neural entrainment occurs independently of somatosensory input (Vieira et al., 2020), human studies indicate that peripheral sensations could still potentially influence sensorimotor oscillations (Asamoah et al., 2019). This raises questions about whether our intensity-dependent effects reflect direct neural modulation, sensory feedback, or a combination of both mechanisms. Future studies should implement enhanced sham protocols with topical anesthetics to minimize sensory differences between conditions (Antal et al., 2017) or utilize active control site stimulation, which is commonly used in the literature (Kasten et al., 2019; Krause et al., 2022), to distinguish between direct neural entrainment and sensory-mediated effects. Clinical implications.

The present findings may have important implications for developing tACS-based interventions for individuals with motor impairments. Sensorimotor beta oscillations show abnormal patterns in various neurological conditions, including stroke (Rossiter et al., 2014), Parkinson’s disease (Heinrichs-Graham et al., 2014), and dystonia (Neumann et al., 2015). Our observation that different tACS intensities produce distinct effects on sensorimotor oscillatory activity provides initial insights for potential applications in these populations. For instance, in Parkinson’s disease, where excessive beta synchronization has been associated with motor symptoms (Little and Brown, 2014), our findings of decreased post-movement power observed following 0.5 mA tACS suggest that this protocol could be used to influence pathological synchronization patterns, though direct testing in patient populations is needed. Notably, the relationship between pre-tACS peak beta frequency and stimulation response suggests individual oscillatory profiles may be important to consider in future protocols. However, important questions remain before clinical translation. Our observed effects were primarily broadband rather than specific to the beta frequency targeted by stimulation, and we cannot yet definitively state whether increasing or decreasing oscillatory power would normalize function in clinical populations. Future research should investigate how these neurophysiological effects translate to functional improvements, whether individualizing stimulation frequencies enhance outcomes, and how protocols might be optimized for different movement phases in specific neurological conditions. With systematic research addressing these questions, tACS may hold promise as a non-invasive approach for addressing motor dysfunction.

### 4.6 Conclusion

The current study examined how different intensities of beta tACS over the left M1 influence sensorimotor oscillatory activity. While none of the tested intensities (0.5 mA, 1.0 mA, or 1.5 mA) effectively modulated resting-state beta power, we observed that tACS intensity had non-linear effects on movement-related power changes. This represents the first systematic investigation of how beta tACS intensity affects sensorimotor power. Our findings highlight the importance of individual peak beta frequency in determining tACS response and provide novel insights into how the motor system responds to beta tACS at different intensities. These results advance our understanding of tACS-induced neuromodulation and have practical implications for optimizing stimulation protocols in both research and clinical applications.

## Data Availability

The raw data supporting the conclusions of this article will be made available by the authors, without undue reservation.
