# Supplementary material for: Beta tACS of varying intensities differentially affect resting-state and movement-related sensorimotor power
Source: Front Neurosci. 2025 Jun 4;19:1524653. doi: 10.3389/fnins.2025.1524653 (PMC12174377; doi:10.3389/fnins.2025.1524653)
Supplement: Supplementary file 1 [file Data_Sheet_1.docx]

Supplementary Material

# Supplementary Methods

## Determining the hand area of M1 for tACS

To determine the sites for tACS electrode placement, surface electromyographic (EMG) recordings and single-pulse TMS were used to identify the left M1 representation of the FDI. EMG activity was recorded from the right FDI using Ag/AgCl cup electrodes in a belly-tendon configuration. The raw electrical signal from the target muscle was amplified 1000 times (CED 1902 amplifier) and band pass filtered (20 – 1000 Hz). The signal was then digitized at a sampling value of 5000 Hz (CED Power1401).

TMS was delivered with Magstim 200^2^ (Magstim, Whitland, Dyfed, UK) via a 90 mm figure-of-eight coil. The coil was placed tangentially to the scalp with the handle pointed backward, 45^o^ away from the midline (that is, at the optimal coil orientation for inducing posterior-anterior current flow in M1; Janssen et al., 2015). The optimal site on the scalp for eliciting motor evoked potentials (MEPs) in the FDI was located using single pulses of TMS. The optimal site was defined as the site that evoked the largest and most reliable MEPs (Rossini et al., 1994, 2015). Once the optimal site was determined, the scalp was marked for placement of the center tACS electrode.

## Electric field models

Electric field simulations of the 4×1 HD-tACS montage were conducted with SimNIBS (v.3.2.0; Thielscher et al., 2015) using finite-element methods. The SimNIBS “headreco” function was used to generate a head model from the MNI152 template brain. For all simulations, electrodes were modeled as rubber discs (conductivity: 29.400 S/m) that were 20 mm in diameter and 2 mm thick, with conductive gel (conductivity: 1.000 S/m) of 2 mm thickness underneath. The center electrode was placed over the C3 coordinate of the 10-20 system, and the four return electrodes were each placed at a radius of 50 mm. Supplementary Table 1 shows the mean electric field strength at left M1, as well as the current focality, for each stimulation intensity. The mean electric field strength at left M1 was computed by means of binary masks, based on the human motor area template (Archer et al., 2018).

**Supplementary Table 1.** Simulated electric field strength and focality

| **Stimulation Intensity** | **Mean Electric Field Strength at Left M1** | **Electric Field Focality** | |
| --- | --- | --- | --- |
|  |  | **Vol_75_** | **Vol_50_** |
| 0.5mA | 2.80E-03 V/m | 7.78E+03 mm^3^ | 2.47E+03 mm^3^ |
| 1.0mA | 5.65E-03 V/m | 7.78E+03 mm^3^ | 2.47E+03 mm^3^ |
| 1.5mA | 8.46E-03 V/m | 7.78E+03 mm^3^ | 2.47E+03 mm^3^ |

# Supplementary Results

**Supplementary Table 2.** Mean (± SD) ratings of specific sensations across stimulation intensities

| **Sensation** | **Stimulation intensity** | | | |
| --- | --- | --- | --- | --- |
|  | sham | 0.5 mA | 1.0 mA | 1.5 mA |
| Itching | 0.725 ± 0.850 | 0.400 ± 0.681 | 0.700 ± 0.865 | 0.700 ± 0.923 |
| Tingling | 0.850 ± 1.182 | 0.550 ± 0.759 | 0.825 ± 0.783 | 1.275 ± 1.272 |
| Headache | 0.000 ± 0.000 | 0.158 ± 0.375 | 0.275 ± 0.638 | 0.100 ± 0.308 |
| Pain in the neck | 0.000 ± 0.000 | 0.000 ± 0.000 | 0.250 ± 0.550 | 0.100 ± 0.447 |
| Paint at the skull | 0.000 ± 0.000 | 0.000 ± 0.000 | 0.000 ± 0.000 | 0.000 ± 0.000 |
| Burning sensation | 0.425 ± 0.748 | 0.100 ± 0.308 | 0.400 ± 0.681 | 0.600 ± 0.940 |
| Heat | 0.500 ± 0.607 | 0.325 ± 0.467 | 0.500 ± 0.761 | 0.775 ± 0.835 |
| Fatigue | 0.316 ± 0.820 | 0.368 ± 0.831 | 0.316 ± 0.749 | 0.600 ± 1.095 |
| Uncomfortable feeling | 0.200 ± 0.410 | 0.150 ± 0.489 | 0.200 ± 0.410 | 0.450 ± 0.686 |
| Iron taste in mouth | 0.050 ± 0.224 | 0.000 ± 0.000 | 0.500 ± 0.827 | 0.000 ± 0.000 |
| Concentration problems | 0.368 ± 0.761 | 0.300 ± 0.571 | 0.000 ± 0.000 | 0.500 ± 0.761 |
| Mood swings | 0.050 ± 0.224 | 0.000 ± 0.000 | 0.000 ± 0.000 | 0.000 ± 0.000 |

Note. Data are expressed as mean (M) ± standard deviation (SD). Participants rated the intensity of their perceived sensations during tACS on a Likert scale ranging from 0 (nothing) to 4 (very strong).

## Resting-state power

### Baseline differences in resting-state power between stimulation intensities

In the main manuscript, a GLMM analysis was performed to examine the effect of tACS on resting-state power. Model estimates were obtained for the fixed effects of INTENSITY (sham, 0.5 mA, 1.0 mA, and 1.5 mA), TIME (pre, post_1_, and post_2_), FREQUENCY (theta, alpha, beta, and gamma), and REGION (C3 cluster, C4 cluster). Results revealed significant differences in power between some of the stimulation intensities at baseline. As the highest level of significant interaction was a four-way interaction between INTENSITY, TIME, FREQUENCY, and REGION, the baseline differences for each frequency band and region have been reported in the following Supplementary Tables. Supplementary Table 3 shows the baseline differences in resting-state power between intensities at the C3 cluster (i.e., left M1). Supplementary Table 4 shows the baseline differences in resting-state power between intensities at the C4 cluster (i.e., right M1).

**Supplementary Table 3.** Baseline differences between stimulation intensities in resting-state power at the C3 cluster

| **Frequency band** | **Intensity comparisons** | ***z*** | ***p*** | **Effect size (*d*)** |
| --- | --- | --- | --- | --- |
| Theta | 1.5mA - 1.0mA | -4.389 | < 0.001^***^ | -0.010 |
|  | 1.5mA - 0.5mA | -3.483 | 0.003^**^ | -0.008 |
|  | 1.5mA - sham | 2.113 | 0.208 | 0.005 |
|  | 1.0mA - 0.5mA | 0.915 | 1.000 | 0.002 |
|  | 1.0mA - sham | 6.442 | < 0.001^***^ | 0.015 |
|  | 0.5mA - sham | 5.553 | < 0.001^***^ | 0.013 |
| Alpha | 1.5mA - 1.0mA | -3.519 | 0.003^**^ | -0.007 |
|  | 1.5mA - 0.5mA | -3.572 | 0.002^**^ | -0.007 |
|  | 1.5mA - sham | 2.028 | 0.255 | 0.004 |
|  | 1.0mA - 0.5mA | -0.042 | 1.000 | < -0.001 |
|  | 1.0mA - sham | 5.498 | < 0.001^***^ | 0.011 |
|  | 0.5mA - sham | 5.557 | < 0.001^***^ | 0.011 |
| Beta | 1.5mA - 1.0mA | -3.253 | 0.007^**^ | -0.008 |
|  | 1.5mA - 0.5mA | -2.426 | 0.092 | -0.006 |
|  | 1.5mA - sham | 1.158 | 1.000 | 0.003 |
|  | 1.0mA - 0.5mA | 0.834 | 1.000 | 0.002 |
|  | 1.0mA - sham | 4.371 | < 0.001^***^ | 0.011 |
|  | 0.5mA - sham | 3.556 | 0.002^**^ | 0.009 |
| Gamma | 1.5mA - 1.0mA | -0.819 | 1.000 | -0.003 |
|  | 1.5mA - 0.5mA | -7.366 | < 0.001^***^ | -0.029 |
|  | 1.5mA - sham | -1.040 | 1.000 | -0.004 |
|  | 1.0mA - 0.5mA | -6.514 | < 0.001^***^ | -0.025 |
|  | 1.0mA - sham | -0.224 | 1.000 | -0.001 |
|  | 0.5mA - sham | 6.253 | < 0.001^***^ | 0.024 |

Note. * *p* < 0.05; ** *p* < 0.01; *** *p* < 0.001.

**Supplementary Table 4.** Baseline differences between stimulation intensities in resting-state power at the C4 cluster

| **Frequency band** | **Intensity comparisons** | ***z*** | ***p*** | **Effect size (*d*)** |
| --- | --- | --- | --- | --- |
| Theta | 1.5mA - 1.0mA | -1.055 | 1.000 | -0.002 |
|  | 1.5mA - 0.5mA | 3.348 | 0.005^**^ | 0.007 |
|  | 1.5mA - sham | 4.556 | < 0.001^***^ | 0.010 |
|  | 1.0mA - 0.5mA | 4.384 | < 0.001^***^ | 0.010 |
|  | 1.0mA - sham | 5.577 | < 0.001^***^ | 0.013 |
|  | 0.5mA - sham | 1.236 | 1.000 | 0.003 |
| Alpha | 1.5mA - 1.0mA | -0.053 | 1.000 | < -0.001 |
|  | 1.5mA - 0.5mA | 4.199 | < 0.001^***^ | 0.008 |
|  | 1.5mA - sham | 6.258 | < 0.001^***^ | 0.012 |
|  | 1.0mA - 0.5mA | 4.231 | < 0.001^***^ | 0.008 |
|  | 1.0mA - sham | 6.279 | < 0.001^***^ | 0.012 |
|  | 0.5mA - sham | 2.094 | 0.218 | 0.004 |
| Beta | 1.5mA - 1.0mA | 3.719 | 0.001^**^ | -0.009 |
|  | 1.5mA - 0.5mA | 3.213 | 0.008^**^ | 0.008 |
|  | 1.5mA - sham | 4.700 | < 0.001^***^ | 0.012 |
|  | 1.0mA - 0.5mA | 6.906 | < 0.001^***^ | 0.017 |
|  | 1.0mA - sham | 8.350 | < 0.001^***^ | 0.020 |
|  | 0.5mA - sham | 1.515 | 0.779 | 0.004 |
| Gamma | 1.5mA - 1.0mA | -8.347 | < 0.001^***^ | -0.034 |
|  | 1.5mA - 0.5mA | -6.487 | < 0.001^***^ | -0.027 |
|  | 1.5mA - sham | -5.733 | < 0.001^***^ | -0.024 |
|  | 1.0mA - 0.5mA | 1.886 | 0.356 | 0.007 |
|  | 1.0mA - sham | 2.570 | 0.061 | 0.010 |
|  | 0.5mA - sham | 0.703 | 1.000 | 0.003 |

Note. * *p* < 0.05; ** *p* < 0.01; *** *p* < 0.001.

### Effects of beta tACS on the resting-state power of the theta, alpha, and gamma bands

The GLMM analysis of resting-state power found a higher-order four-way INTENSITY × TIME × FREQUENCY × REGION interaction (χ*^2^* (18, *N* = 21) = 70.20, *p* < 0.001). Post-hoc analyses of resting-state beta power were presented in the main manuscript. Here, we focus on describing the post-hoc analyses of resting-state theta, alpha, and gamma power.

#### Theta Power

As shown in Supplementary Figure 1A, all four stimulation intensities showed increased theta power at the C3 cluster, from pre to post_1_ (|*z*s| ≥ 3.321, *p*s ≤ 0.003, |*d*s| ≥ 0.007) and from pre to post_2_ (|*z*s| ≥ 4.711, *p*s < 0.001, |*d*s| ≥ 0.010). Most stimulation intensities did not show a significant change from post_1_ to post_2_ (|*z*s| ≤ 2.004, *p*s ≥ 0.111, |*d*s| ≤ 0.004), with only 1.5 mA stimulation showing a significant change – a further increase – in theta power (*z* = 2.366, *p* = 0.047, *d* = 0.005). The increases in theta power were unlikely due to tACS, as the extent of changes following real stimulation were not greater than the changes following sham stimulation (|*z*s| ≤ 2.606, *p*s ≥ 0.153, |*d*s| ≤ 0.047). Interestingly, the extent of increase in theta power from pre to post_1_ was greater following sham stimulation than following 0.5 mA stimulation (*z* = 3.339, *p* = 0.015, *d* = 0.060). This finding suggests that 0.5 mA stimulation induced some suppression of theta power.

Supplementary Figure 1B shows the changes in resting-state theta power at the C4 cluster. Results revealed region-specific changes in theta power following 1.0 mA and 1.5 mA stimulation. However, sham stimulation also showed significant region-specific changes, indicating that these regional differences were unlikely related to tACS. From pre to post_1_, sham, 1.0 mA, and 1.5mA stimulation showed significantly greater increases in theta power at the C3 cluster compared to the C4 cluster (|*z*s| ≥ 2.672, *p*s ≤ 0.023, |*d*s| ≥ 0.046). From pre to post_2_, only the sham and 1.5 mA conditions continued to show significantly greater increases around C3 compared to C4 (|*z*s| ≥ 2.521, *p*s ≤ 0.035, |*d*s| ≥ 0.046), while 0.5 mA stimulation showed a significantly greater increase around C4 compared to C3 (*z* = 3.259, *p* = 0.003, *d* = 0.058). From post_1_ to post_2_, there were no differences between the C3 and C4 clusters following any stimulation intensity (|*z*s| ≤ 1.271, *p*s ≥ 0.495, |*d*s| ≤ 0.022).

**
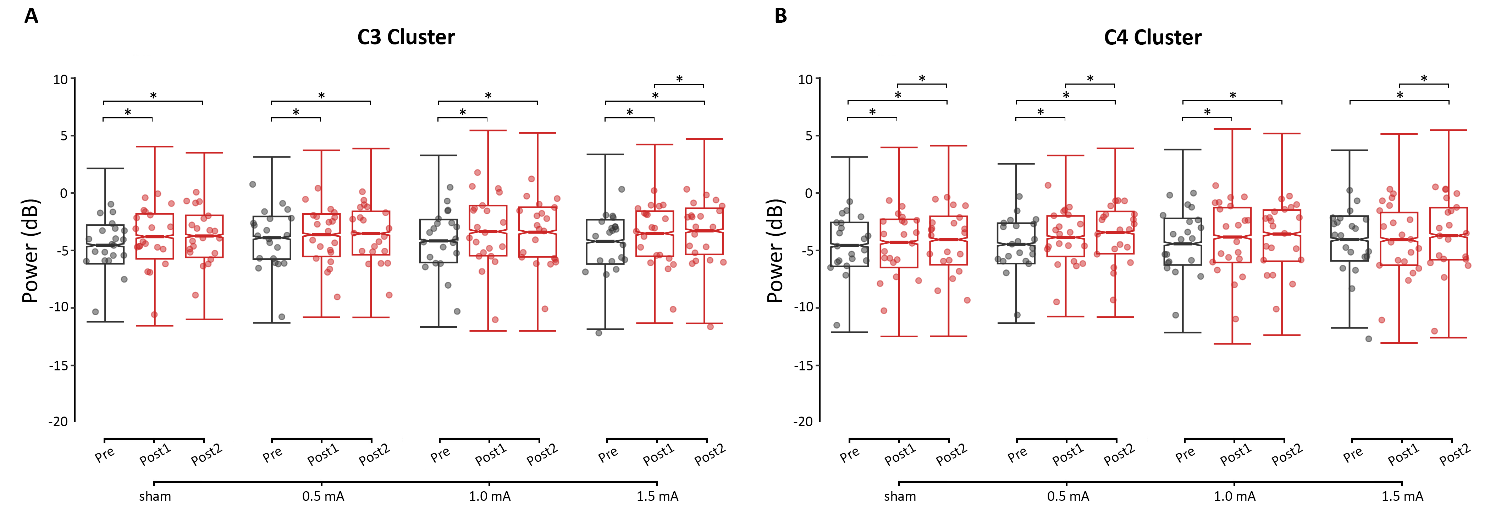
Supplementary Figure 1.** Changes in resting-state theta power for each time-point and stimulation intensity, for (A) the C3 cluster (i.e., left M1) and (B) the C4 cluster (i.e., right M1).* = significant change between time-points at α = 0.05. Data points reflect participant averages. The height of the notches reflects the median +/- 1.57 x IQR/sqrt(n) where IQR is the interquartile range defined by the 25th and 75th percentiles and n is the number of data points.

#### Alpha Power

The changes in resting-state alpha power were comparable to the changes in theta power. As shown in Supplementary Figure 2A, all four stimulation intensities, including sham, showed an increase in alpha power at the C3 cluster, from pre to post_1_ (|*z*s| ≥ 8.215, *p*s < 0.001, |*d*s| ≥ 0.023) and from pre to post_2_ (|*z*s| ≥ 7.553, *p*s < 0.001, |*d*s| ≥ 0.014). However, none of the stimulation intensities showed significant changes in alpha power from post_1_ to post_2_ (|*z*s| ≤ 2.551, *p*s ≥ 0.177, |*d*s| ≤ 0.038). Further, these increases in power were unlikely due to tACS, as the extent of changes following real stimulation were not greater than the changes following sham stimulation (|*z*s| ≤ 3.371, *p*s ≥ 0.013, |*d*s| ≤ 0.051), suggesting that these increases in alpha power were unlikely to have been induced by tACS. Additionally, the increase in alpha power from pre to post_2_ was greater for sham than for 0.5 mA stimulation (*z* = 3.371, *p* = 0.013, *d* = 0.051).

Supplementary Figure 2B shows the changes in resting-state alpha power at the C4 cluster. As with the changes in theta power, region-specific changes in alpha power were observed following both 1.0 mA and 1.5 mA stimulation (|*z*s| ≥ 2.479, *p*s ≤ 0.039, |*d*s| ≥ 0.036), but not 0.5 mA stimulation (|*z*s| ≤ 1.940, *p*s ≥ 0.149, |*d*s| ≤ 0.029). However, contrasting with the changes in theta power, sham stimulation did not show any region-specific changes in alpha power (|*z*s| ≤ 2.227, *p*s ≥ 0.076, |*d*s| ≤ 0.034), indicating that these regional differences in alpha following 1.0 mA and 1.5 mA stimulation might have been related to tACS. From pre to post_1_ and pre to post_2_, 1.0 mA and 1.5 mA stimulation showed significantly greater increases in alpha power at the C3 cluster compared to the C4 cluster. From post_1_ to post_2_, there were no differences between the C3 and C4 clusters following any stimulation intensity (|*z*s| ≤ 0.806, *p*s ≥ 0.805, |*d*s| ≤ 0.012).

***
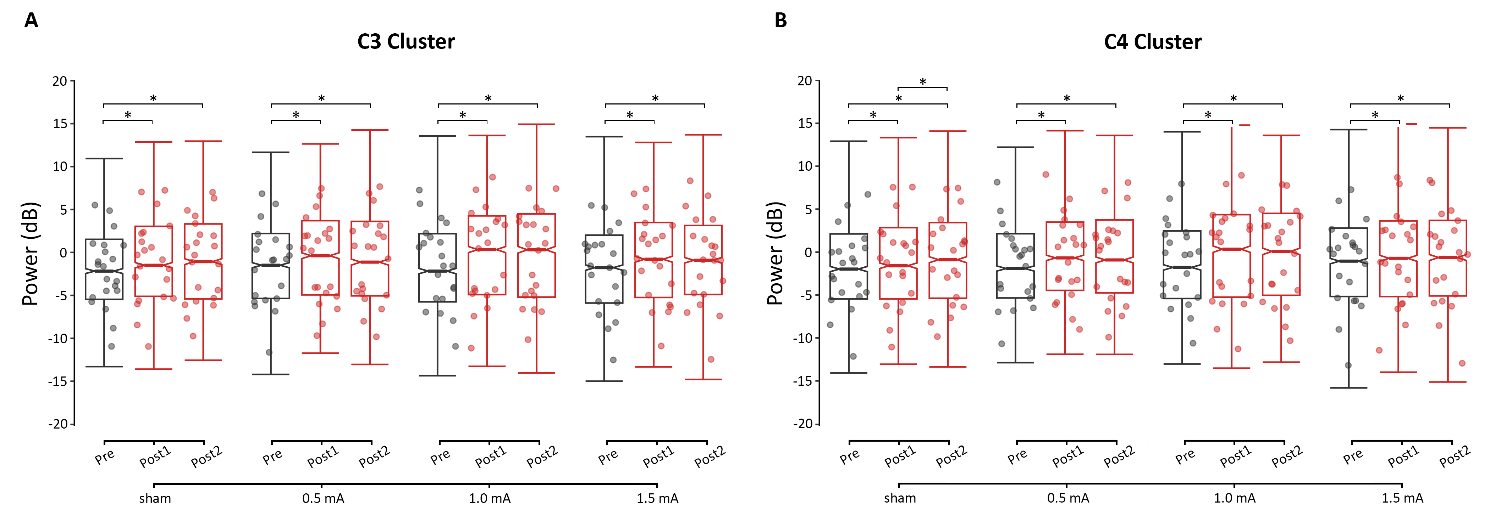
*
Supplementary Figure 2.** Changes in resting-state alpha power for each time-point and stimulation intensity, for (A) the C3 cluster (i.e., left M1) and (B) the C4 cluster (i.e., right M1).* = significant change between time-points at α = 0.05. Data points reflect participant averages. The height of the notches reflects the median +/- 1.57 x IQR/sqrt(n) where IQR is the interquartile range defined by the 25th and 75th percentiles and n is the number of data points.

#### Gamma Power

As shown in Supplementary Figure 3A, the changes in gamma power differed from the other frequency bands – only sham and 0.5 mA stimulation showed significant changes in gamma power at the C3 cluster. Sham stimulation did not show any significant change from pre to post_1_ (*z* = 1.301, *p* = 0.395, *d* = 0.005), but showed significant decreases in C3 gamma power from pre to post_2_ (*z* = 4.617, *p* < 0.001, *d* = 0.019) and from post_1_ to post_2_ (*z* = 5.994, *p* < 0.001, *d* = 0.024). In contrast, 0.5 mA stimulation showed an initial decrease in C3 gamma power (pre to post_1_: *z* = 6.791, *p* < 0.001, *d* = 0.026), which changed to a significant increase in gamma power at post_2_ (pre to post_2_: *z* = 3.391, *p* = 0.002, *d* = 0.012, post_1_ to post_2_: *z* = 10.161, *p* < 0.001, *d* = 0.038). There were no significant changes in C3 gamma power following 1.0 mA nor 1.5 mA stimulation (|*z*s| ≤ 2.126, *p*s ≥ 0.085, |*d*s| ≤ 0.008). Together, these results show that: (1) there was a decrease in C3 gamma power unrelated to tACS, (2) 20 Hz tACS might have induced an intensity-dependent effect on C3 gamma power – 1.0 mA and 1.5 mA tACS might have prevented the decrease of gamma power seen following sham, and 0.5 mA tACS led to an increase in C3 gamma power.

Supplementary Figure 3B shows the changes in resting-state gamma power at the C4 cluster. Region-specific changes in gamma power were also observed from pre to post_1_ and from post_1_ to post_2_, following sham and 1.0 mA stimulation. From pre to post_1_, both sham and 1.0 mA stimulation showed significant decreases in gamma power at the C4 cluster and unchanged levels of gamma power at the C3 cluster (|*z*s| ≥ 6.891, *p*s < 0.001, |*d*s| ≥ 0.229). As sham and 1.0 mA stimulation showed a comparable pattern of difference between the C3 and C4 clusters, it is unlikely that any of these differences from pre to post1 were related to tACS. From post_1_ to post_2_, sham stimulation showed a significant decrease in the C3 but not the C4 cluster (*z* = 4.641, *p* < 0.001, *d* = 0.158). In contrast, 1.0 mA stimulation showed a significant increase in the C4 but not the C3 cluster (*z* = 8.112, *p* < 0.001, *d* = 0.273). There were no other region-specific changes (|*z*s| ≤ 2.095, *p*s ≥ 0.105, |*d*s| ≤ 0.071).

**
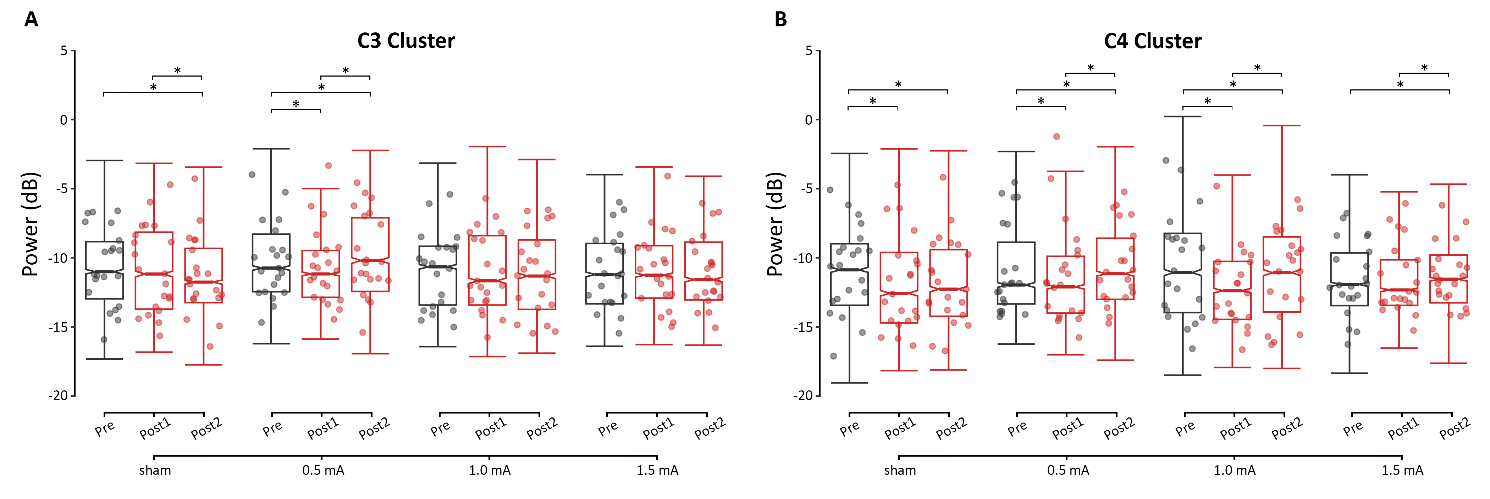

Supplementary Figure 3.** Changes in resting-state gamma power for each time-point and stimulation intensity, for (A) the C3 cluster (i.e., left M1) and (B) the C4 cluster (i.e., right M1).* = significant change between time-points at α = 0.05. Data points reflect participant averages. The height of the notches reflects the median +/- 1.57 x IQR/sqrt(n) where IQR is the interquartile range defined by the 25th and 75th percentiles and n is the number of data points.

## Event-related power

### Baseline differences in event-related power between stimulation intensities

#### Pre-movement period (-500 – 0 ms)

In the main manuscript, a GLMM analysis was performed to examine the effect of tACS on the event-related power of the pre-movement period. Model estimates were obtained for the fixed effects of INTENSITY, TIME, FREQUENCY, and REGION. Results revealed significant differences in power between some of the stimulation intensities at baseline. As the highest level of significant interaction was a two-way interaction between INTENSITY and TIME, the baseline differences in bilateral broadband (4 – 90 Hz) power of the pre-movement period have been reported in Supplementary Table 5.

**Supplementary Table 5.** Baseline differences between stimulation intensities in the bilateral broadband (4 – 90 Hz) power of the pre-movement period

| **Intensity comparisons** | ***z*** | ***p*** | **Effect size (*d*)** |
| --- | --- | --- | --- |
| 1.5mA - 1.0mA | 1.564 | 0.707 | 0.001 |
| 1.5mA - 0.5mA | 0.836 | 1.000 | 0.001 |
| 1.5mA - sham | -1.703 | 0.531 | -0.002 |
| 1.0mA - 0.5mA | -0.74 | 1.000 | -0.001 |
| 1.0mA - sham | -3.279 | 0.006^**^ | -0.003 |
| 0.5mA - sham | -2.56 | 0.063 | -0.002 |

#### Movement period (0 – 500 ms)

A GLMM analysis was also performed to examine the effect of tACS on the event-related power of the pre-movement period. Model estimates were obtained for the fixed effects of INTENSITY, TIME, FREQUENCY, and REGION. Results revealed significant differences in power between some of the stimulation intensities at baseline. As the highest level of significant interaction was a three-way interaction between INTENSITY, TIME, and FREQUENCY, the baseline differences in the bilateral power have been reported for each frequency band of the movement period in Supplementary Table 6.

**Supplementary Table 6.** Baseline differences between stimulation intensities in the bilateral power of the movement period

| **Frequency band** | **Intensity comparisons** | ***z*** | ***p*** | **Effect size (*d*)** |
| --- | --- | --- | --- | --- |
| Theta | 1.5mA - 1.0mA | 2.072 | 0.162 | 0.004 |
|  | 1.5mA - 0.5mA | 3.185 | 0.008^**^ | 0.006 |
|  | 1.5mA - sham | 1.772 | 0.287 | 0.003 |
|  | 1.0mA - 0.5mA | 1.094 | 0.693 | 0.002 |
|  | 1.0mA - sham | -0.319 | 0.989 | -0.001 |
|  | 0.5mA - sham | -1.426 | 0.483 | -0.003 |
| Alpha | 1.5mA - 1.0mA | 1.81 | 0.268 | 0.003 |
|  | 1.5mA - 0.5mA | 2.147 | 0.139 | 0.004 |
|  | 1.5mA - sham | -1.893 | 0.231 | -0.004 |
|  | 1.0mA - 0.5mA | 0.32 | 0.989 | 0.001 |
|  | 1.0mA - sham | -3.716 | 0.001^**^ | -0.007 |
|  | 0.5mA - sham | -4.073 | < 0.001^***^ | -0.008 |
| Beta | 1.5mA - 1.0mA | 0.058 | 1.000 | < 0.001 |
|  | 1.5mA - 0.5mA | 0.012 | 1.000 | < 0.001 |
|  | 1.5mA - sham | -0.695 | 0.899 | -0.001 |
|  | 1.0mA - 0.5mA | -0.047 | 1.000 | < -0.001 |
|  | 1.0mA - sham | -0.753 | 0.876 | -0.001 |
|  | 0.5mA - sham | -0.712 | 0.892 | -0.001 |
| Gamma | 1.5mA - 1.0mA | -0.56 | 0.944 | -0.001 |
|  | 1.5mA - 0.5mA | 0.497 | 0.960 | 0.001 |
|  | 1.5mA - sham | -0.343 | 0.986 | -0.001 |
|  | 1.0mA - 0.5mA | 1.06 | 0.714 | 0.002 |
|  | 1.0mA - sham | 0.222 | 0.996 | < 0.001 |
|  | 0.5mA - sham | -0.847 | 0.832 | -0.002 |

Note. * *p* < 0.05; ** *p* < 0.01; *** *p* < 0.001.

#### Post-movement period (1500 – 4000 ms)

A GLMM analysis was performed to examine the effect of tACS on the event-related power of the post-movement period. Model estimates were obtained for the fixed effects of INTENSITY, TIME, FREQUENCY, and REGION. Results revealed no significant differences in power between stimulation intensities at baseline. As the highest level of significant interaction was a two-way interaction between INTENSITY and TIME, the baseline differences in bilateral broadband (4 – 90 Hz) power of the post-movement period have been reported in Supplementary Table 7.

**Supplementary Table 7.** Baseline differences between stimulation intensities in the bilateral broadband (4 – 90 Hz) power of the post-movement period

| **Intensity comparisons** | ***z*** | ***p*** | **Effect size (*d*)** |
| --- | --- | --- | --- |
| 1.5mA - 1.0mA | -0.111 | 1.000 | < -0.001 |
| 1.5mA - 0.5mA | -1.498 | 0.439 | -0.001 |
| 1.5mA - sham | -2.162 | 0.134 | -0.002 |
| 1.0mA - 0.5mA | -1.385 | 0.509 | -0.001 |
| 1.0mA - sham | -2.048 | 0.170 | -0.002 |
| 0.5mA - sham | -0.667 | 0.909 | -0.001 |

### Effects of beta tACS on the event-related power of the movement period (0 – 500 ms), in the theta, alpha, and gamma bands

The GLMM analysis of event-related power of the movement period found a significant three-way interaction of INTENSITY, TIME, and FREQUENCY (*χ*^2^ (18, *N* = 20) = 30.83, *p* = 0.030). This was not mediated by a higher-level four-way interaction of INTENSITY, TIME, FREQUENCY, and REGION (*χ*^2^ (18, *N* = 20) = 9.14, *p* = 0.956). Post-hoc analyses of event-related beta power were presented in the main manuscript. Here, we focus on describing the post-hoc analyses of event-related theta, alpha, and gamma power.

#### Theta power

As shown in the Supplementary Figure 4, a change in theta power was only observed from post_1_ to post_2_. Between these time points, theta power decreased following sham stimulation (*z* = 3.572, *p* = 0.001, *d* = 0.007), which may have been a chance occurrence (due to noise) or may indicate that the real stimulation intensities promoted stability in event-related theta power.


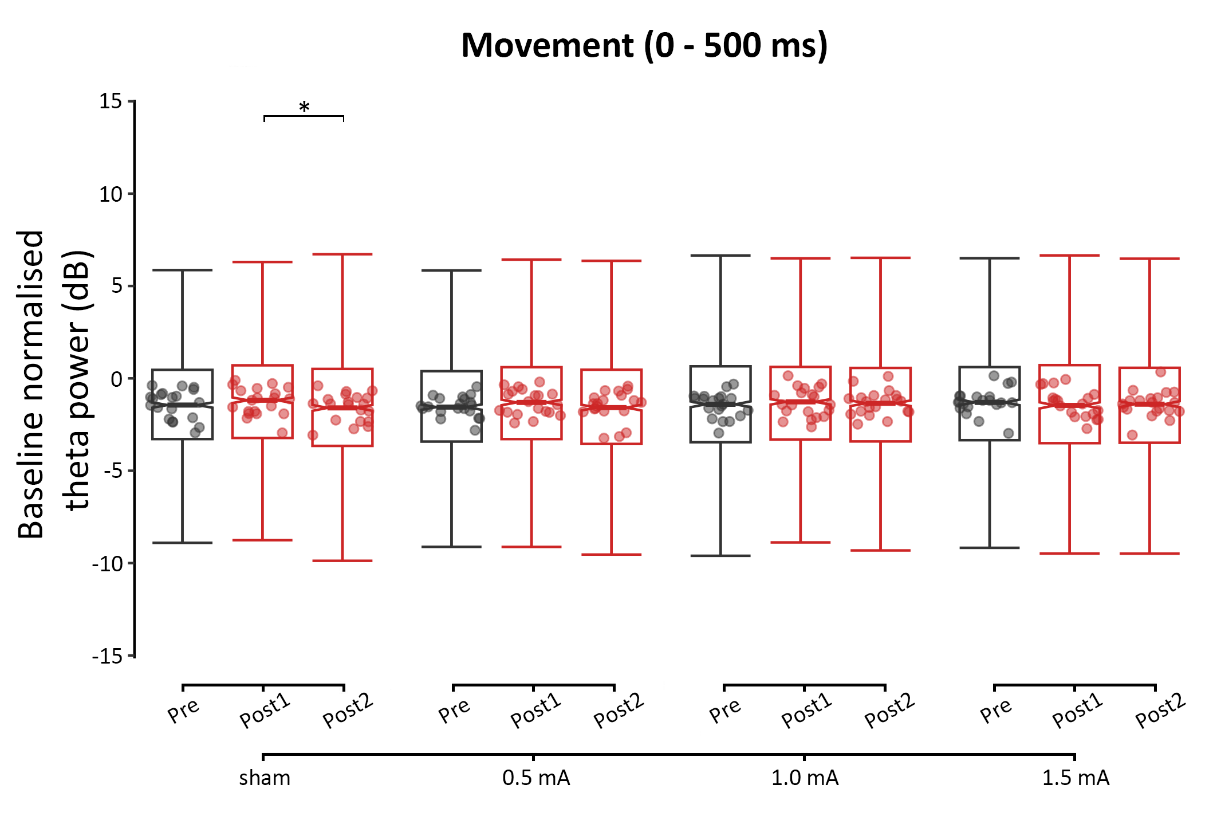


**Supplementary Figure 4.** Event-related changes bilateral theta power (4 to 7 Hz) of the movement period (0 to 500 ms), for each stimulation intensity. Power values have been baseline-normalized to the period of -2000 to -1000 ms. Data points reflect participant averages. The height of the notches reflects the median +/- 1.57 x IQR/sqrt(n) where IQR is the interquartile range defined by the 25th and 75th percentiles and n is the number of data points.

#### Alpha power

As shown in the Supplementary Figure 5, there was a decrease in alpha power from pre to post_1_ following sham stimulation (*z* = 3.996, *p* < 0.001, *d* = 0.008). From pre to post_2_, there were decreases in alpha power following sham, 0.5 mA, and 1.5 mA stimulation (|*z*s| ≥ 2.819, *p*s ≤ 0.013, |*d*s| ≥ 0.006). It is unlikely that these decreases in alpha power were due to tACS, as none of the real stimulation intensities showed greater decreases in alpha power relative to sham stimulation (|*z*s| ≤ 0.660, *p*s = 1.000, |*d*s| ≤ 0.058). From post_1_ to post_2_, there was a decrease in alpha power only following 0.5 mA stimulation (*z* = 3.613, *p* < 0.001, *d* = 0.007). As this was the only stimulation intensity to show a decrease in alpha power from post_1_ to post_2_, it is possible that this reflected an intensity-specific suppression in event-related alpha power. Interestingly, there was one stimulation intensity that did not show any decrease in event-related alpha power – 1.0 mA (|*z*s| ≤ 1.819, *p*s ≥ 0.163, |*d*s| ≤ 0.003). This might suggest that 1.0 mA stimulation promoted stability in event-related alpha power.

**
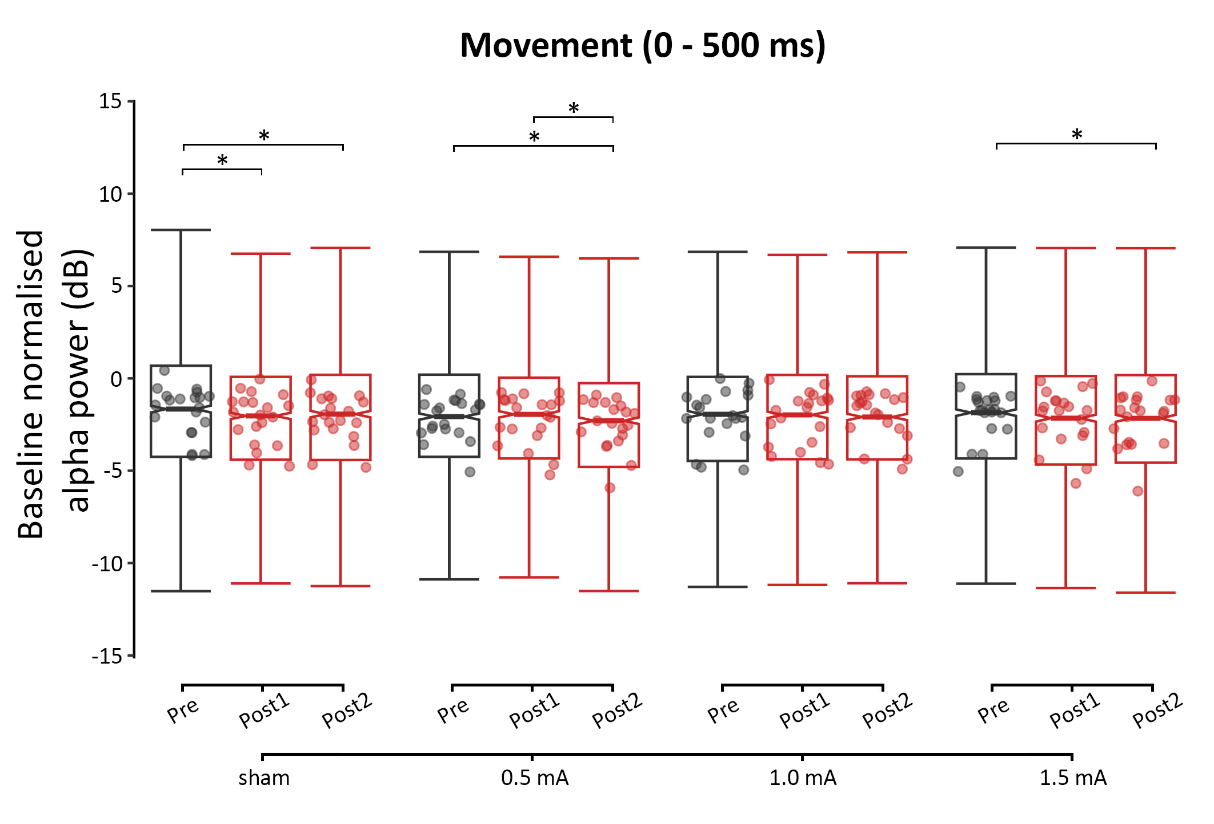
**

**Supplementary Figure 5.** Event-related changes bilateral alpha power (8 to 12 Hz) of the movement period (0 to 500 ms), for each stimulation intensity. Power values have been baseline-normalized to the period of -2000 to -1000 ms. Data points reflect participant averages. The height of the notches reflects the median +/- 1.57 x IQR/sqrt(n) where IQR is the interquartile range defined by the 25th and 75th percentiles and n is the number of data points.

#### Gamma power

As shown in the Supplementary Figure 6, no changes in gamma power were observed following any of the stimulation intensities (|*z*s| ≤ 1.678, *p*s ≥ 0.280, |*d*s| ≤ 0.003).

**
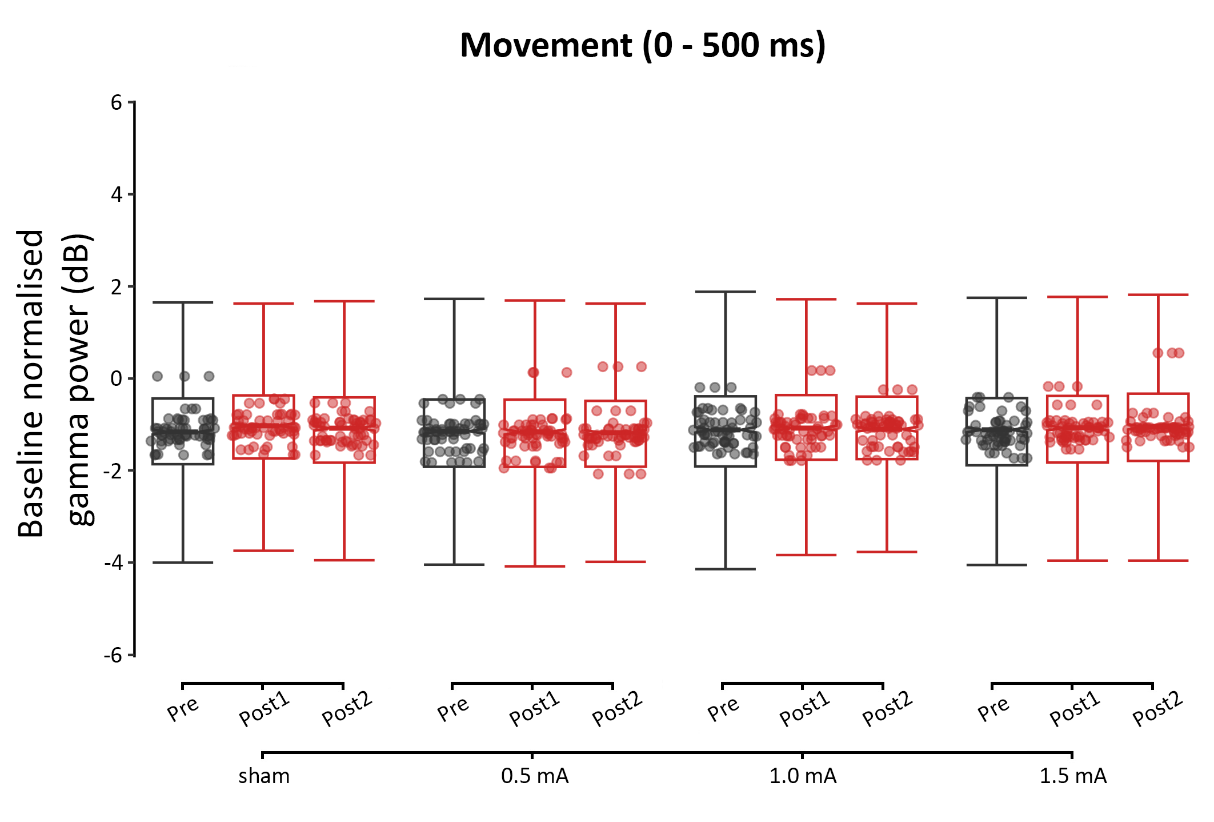
**

**Supplementary Figure 6.** Event-related changes bilateral gamma power (60 to 90 Hz) of the movement period (0 to 500 ms), for each stimulation intensity. Power values have been baseline-normalized to the period of -2000 to -1000 ms. Data points reflect participant averages. The height of the notches reflects the median +/- 1.57 x IQR/sqrt(n) where IQR is the interquartile range defined by the 25th and 75th percentiles and n is the number of data points.

# Supplementary Discussion

## Between session differences in baseline sensorimotor power

We observed between-session differences in baseline sensorimotor power, present in both resting-state and event-related data. There are two potential explanations for these findings. First, between-session differences could be attributed to the inherent test-retest reliability challenges associated with EEG power measurements. These measurements are sensitive to various technical and physiological factors that can vary from session to session, such as the signal-to-noise ratio (Holler et al., 2017; Kappenman & Luck, 2010). Second, these variations might reflect natural intra-individual variability in neural activity. Importantly, the GLMMs accounted for this variability by incorporating participant-specific random intercepts, allowing for appropriate modeling of individual differences.

## Cross-frequency changes in sensorimotor power

We observed intensity-dependent modulations of resting-state power theta and gamma power at left M1. 0.5 mA stimulation led to a decrease in theta and gamma power at post1, followed by an increase in gamma power at post2 that surpassed baseline levels. In contrast, there were no changes in resting-state theta or gamma power following 1.0 mA and 1.5 mA stimulation, and no cross-frequency modulations of event-related power of the movement period. These findings indicate that beta tACS interacted with resting-state, but not movement-related, oscillations outside of the target frequency band. To our knowledge, this is the first report of change in resting-state theta and gamma power following left M1 beta tACS, as most relevant studies have limited their analyses to the targeted frequency band (Lafleur et al., 2021; Rumpf et al., 2019; Suzuki et al., 2022). These changes might reflect cross-frequency phase-amplitude coupling (Canolty & Knight, 2010; Hyafil et al., 2015; Jensen & Colgin, 2007), or have resulted from changes in the excitation/inhibition balance (Michalareas et al., 2016). Indeed, several studies have reported cross-frequency changes in oscillations following tACS of other frequencies (e.g., de la Salle et al., 2023; Helfrich et al., 2016; Jones et al., 2020).

It is unclear why cross-frequency modulation differed between brain states, though this finding aligns with previous research. Multiple studies have demonstrated that tACS effects are state-dependent (e.g., Fiene et al., 2020; Nguyen et al., 2018; Ozen et al., 2010; Shorafa et al., 2021; Wang et al., 2022). Such state-based differences in tACS response likely stem from underlying variations in neurophysiological parameters, such as the excitation/inhibition balance (Francis et al., 2003), which can significantly influence how neural tissue responds to electrical stimulation. It is also unclear why only resting-state theta and gamma oscillations were modulated by beta tACS (compared to alpha). The modulation of gamma might be explained by the functional interaction between theta, beta and gamma within the motor network: both beta-gamma and theta-gamma coupling have been suggested to play an important role in motor control (De Hemptinne et al., 2013; Gong et al., 2022; Spooner & Wilson, 2022). Alternatively, this result might reflect a feedback loop, where beta tACS altered the balance of excitatory and inhibitory circuits, leading to changes in theta and gamma oscillations (Michalareas et al., 2016).

Taken together, these findings demonstrate that beta tACS over left M1 can induce intensity-dependent cross-frequency modulations in resting-state theta and gamma power, with effects being specific to lower stimulation intensities (0.5 mA) and brain state (rest vs. movement). These results highlight the complex interactions between stimulation parameters, brain state, and oscillatory dynamics in the motor network.

# Supplementary References

Canolty, R. T., and Knight, R. T. (2010). The functional role of cross-frequency coupling. *Trends Cogn Sci* 14, , 1–21. doi: 10.1016/J.TICS.2010.09.001

De Hemptinne, C., Ryapolova-Webb, E. S., Air, E. L., Garcia, P. A., Miller, K. J., Ojemann, J. G., et al. (2013). Exaggerated phase-amplitude coupling in the primary motor cortex in Parkinson disease. *Proc Natl Acad Sci* 110, 4780–4785. doi: 10.1073/PNAS.1214546110

de la Salle, S., Choueiry, J., Payumo, M., Devlin, M., Noel, C., Abozmal, A., et al. (2023). Transcranial alternating current stimulation alters auditory steady-state oscillatory rhythms and their cross-frequency couplings. *Clin EEG Neurosci* 55(3), 329–339. doi: 10.1177/15500594231179679

Fiene, M., Schwab, B. C., Misselhorn, J., Herrmann, C. S., Schneider, T. R., and Engel, A. K. (2020). Phase-specific manipulation of rhythmic brain activity by transcranial alternating current stimulation. *Brain Stimul* 13(5), 1254–1262. doi:10.1016/j.brs.2020.06.008

Francis, J. T., Gluckman, B. J., and Schiff, S. J. (2003). Sensitivity of neurons to weak electric fields. *J Neuro* 23(19), 7255–7261. doi: 10.1523/JNEUROSCI.23-19-07255.2003

Gong, R., Muhlberg, C., Wegscheider, M., Fricke, C., Rumpf, J. J., Knosche, T. R., et al. (2022). Cross-frequency phase-amplitude coupling in repetitive movements in patients with Parkinson’s disease. *J Neurophysiol* 127, 1606–1621. doi: 10.1152/jn.00541.2021

Helfrich, R. F., Herrmann, C. S., Engel, A. K., and Schneider, T. R. (2016). Different coupling modes mediate cortical cross-frequency interactions. *Neuroimage* 140, 76–82. doi: 10.1016/J.NEUROIMAGE.2015.11.035

Höller, Y., Uhl, A., Bathke, A., Thomschewski, A., Butz, K., Nardone, R., Fell, J., and Trinka, E. (2017) Reliability of EEG measures of interaction: A paradigm shift is needed to fight the reproducibility crisis. *Front Hum Neurosci* 11, 1–15. doi: 10.3389/fnhum.2017.00441

Hyafil, A., Giraud, A. L., Fontolan, L., and Gutkin, B. (2015). Neural cross-frequency coupling: Connecting architectures, mechanisms, and functions. *Trends Neurosci* 38, 725–740. doi: 10.1016/J.TINS.2015.09.001

Lafleur, L.-P., Murray, A., Desforges, M., Pacheco-Barrios, K., Fregni, F., Tremblay, S., et al. (2021). No aftereffects of high current density 10 Hz and 20 Hz tACS on sensorimotor alpha and beta oscillations. *Sci Rep* 11, 1–10. doi: 10.1038/s41598-021-00850-1

Jensen, O., and Colgin, L. L. (2007). Cross-frequency coupling between neuronal oscillations. *Trends Cogn Sci* 11, 267–269. doi: 10.1016/J.TICS.2007.05.003

Jones, K. T., Johnso, E. L., Taux, Z. S., and Roja, D. C. (2020). Modulation of auditory gamma-band responses using transcranial electrical stimulation. *J Neurophysiol* 123, 2504–2514. doi: 10.1152/jn.00003.2020

Kappenman, E. S., Luck, S. J. (2010). The effects of electrode impedance on data quality and statistical significance in ERP recordings. *Psychophysiology* 47(5) 888–904. doi: 10.1111/j.1469-8986.2010.01009

Michalareas, G., Vezoli, J., van Pelt, S., Schoffelen, J. M., Kennedy, H., and Fries, P. (2016). Alpha-beta and gamma rhythms subserve feedback and feedforward influences among human visual cortical areas. *Neuron* 89, 384–397. doi: 10.1016/J.NEURON.2015.12.018

Nguyen, J., Deng, Y., and Reinhart, R. M. G. (2018). Brain-state determines learning improvements after transcranial alternating-current stimulation to frontal cortex. *Brain Stimul* 11(4), 723–726. doi: 10.1016/j.brs.2018.02.008

Ozen, S., Sirota, A., Belluscio, M. A., Anastassiou, C. A., Stark, E., Koch, C., and Buzsáki, G. (2010). Transcranial electric stimulation entrains cortical neuronal populations in rats. *J Neurosci* 30(34), 11476–11485. doi: 10.1523/JNEUROSCI.5252-09.2010

Rumpf, J.-J., Barbu, A., Fricke, C., Wegscheider, M., and Classen, J. (2019). Posttraining alpha transcranial alternating current stimulation impairs motor consolidation in elderly people. *Neural Plast* 1–11. doi: 10.1155/2019/2689790

Shorafa, Y., Halawa, I., Hewitt, M., Nitsche, M. A., Antal, A., and Paulus, W. (2021). Isometric agonist and antagonist muscle activation interacts differently with 140-Hz transcranial alternating current stimulation aftereffects at different intensities. *J Neurophysiol* 126, 340–348. doi: 10.1152/jn.00065.2021

Spooner, R. K., and Wilson, T. W. (2022) Cortical theta–gamma coupling governs the adaptive control of motor commands. *Brain Commun* 4(6), 1–12. doi: 10.1093/braincomms/fcac249

Suzuki, M., Tanaka, S., Gomez-Tames, J., Okabe, T., Cho, K., Iso, N., et al. (2022). Nonequivalent after-effects of alternating current stimulation on motor cortex oscillation and inhibition: simulation and experimental study. *Brain Sci* 12, 1–15. doi: 10.3390/BRAINSCI12020195

Wang, Y., Hou, P., Li, W., Zhang, M., Zheng, H., and Chen, X. (2022). The influence of different current-intensity transcranial alternating current stimulation on the eyes-open and eyes-closed resting-state electroencephalography. *Front Hum Neurosci* 16, 1–10. doi: 10.3389/fnhum.2022.934382
